# *Corylus avellana* L. Aroma Blueprint: Potent Odorants Signatures in the Volatilome of High Quality Hazelnuts

**DOI:** 10.3389/fpls.2022.840028

**Published:** 2022-03-03

**Authors:** Simone Squara, Federico Stilo, Marta Cialiè Rosso, Erica Liberto, Nicola Spigolon, Giuseppe Genova, Giuseppe Castello, Carlo Bicchi, Chiara Cordero

**Affiliations:** ^1^Dipartimento di Scienza e Tecnologia del Farmaco, Università degli Studi di Torino, Turin, Italy; ^2^Laemmegroup - A Tentamus Company, Turin, Italy; ^3^Soremartec Italia Srl, Cuneo, Italy

**Keywords:** *Corylus avellana L*., key-aroma compounds, metabolomics, chromatographic fingerprinting, hazelnuts volatilome, primary metabolites, volatile organic compounds (V.O.C.)

## Abstract

The volatilome of hazelnuts (*Corylus avellana* L.) encrypts information about phenotype expression as a function of cultivar/origin, post-harvest practices, and their impact on primary metabolome, storage conditions and shelf-life, spoilage, and quality deterioration. Moreover, within the bulk of detectable volatiles, just a few of them play a key role in defining distinctive aroma (i.e., aroma blueprint) and conferring characteristic hedonic profile. In particular, in raw hazelnuts, key-odorants as defined by *sensomics* are: 2,3-diethyl-5-methylpyrazine (*musty* and *nutty*); 2-acetyl-1,4,5,6-tetrahydropyridine (*caramel*); 2-acetyl-1-pyrroline (*popcorn-like*); 2-acetyl-3,4,5,6-tetrahydropyridine (*roasted, caramel*); 3-(methylthio)-propanal (*cooked potato*); 3-(methylthio)propionaldehyde (*musty, earthy*); 3,7-dimethylocta-1,6-dien-3-ol/linalool (citrus, floral); 3-methyl-4-heptanone (*fruity, nutty*); and 5-methyl-(E)-2-hepten-4-one (*nutty, fruity*). Dry-roasting on hazelnut kernels triggers the formation of additional potent odorants, likely contributing to the pleasant aroma of roasted nuts. Whiting the newly formed aromas, 2,3-pentanedione (*buttery*); 2-propionyl-1-pyrroline (*popcorn-like*); 3-methylbutanal; (*malty*); 4-hydroxy-2,5-dimethyl-3(2H)-furanone (*caramel*); dimethyl trisulfide (*sulfurous, cabbage*) are worthy to be mentioned. The review focuses on high-quality hazelnuts adopted as premium primary material by the confectionery industry. Information on primary and secondary/specialized metabolites distribution introduces more specialized sections focused on volatilome chemical dimensions and their correlation to cultivar/origin, post-harvest practices and storage, and spoilage phenomena. Sensory-driven studies, based on *sensomic* principles, provide insights on the aroma blueprint of raw and roasted hazelnuts while robust correlations between non-volatile precursors and key-aroma compounds pose solid foundations to the conceptualization of *aroma potential*.

## Introduction

European hazelnut (*Corylus avellana* L.) belongs to the *Corylus* genus, Betulaceae birch family, and is one of the 25 existing hazelnut species (Erdogan and Mehlenbacher, 2000); originally of the Black sea region, *C. avellana* L. has been cultivated since Roman times (Boccacci and Botta, 2009), but intensive production started to expand in the 1930s in the Langhe region in Piemonte (North-West of Italy) due to the demand from the confectionery industry, and since 1964 in Turkey (Bozoglu, 2005). *C. avellana* L. is the main species of interest for industrial applications due to high-quality characteristics such as larger kernels and thinner shells (Erdogan and Mehlenbacher, 2000).

**Figure 1:**
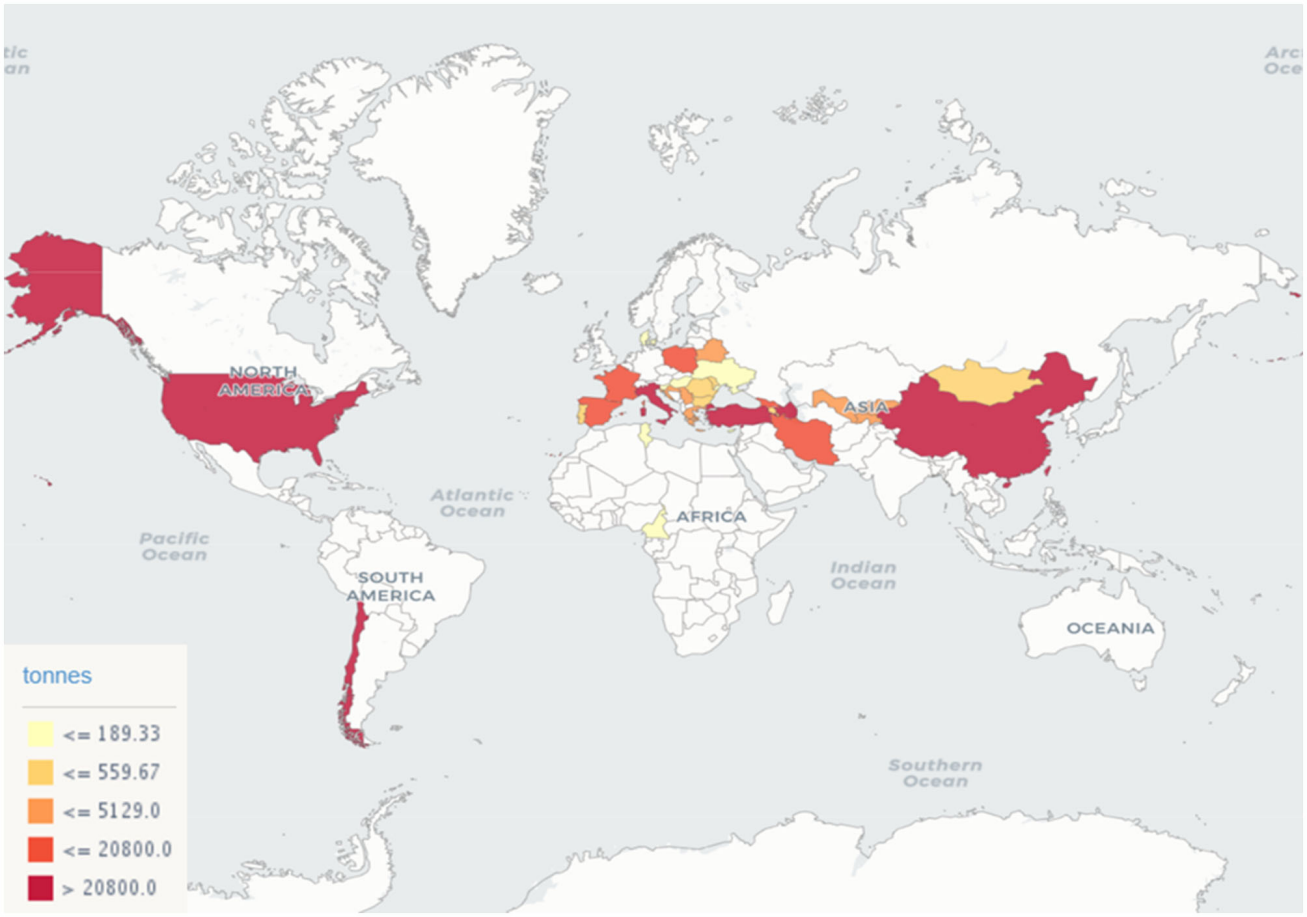
In-shell hazelnut production during the years 2017-2019 according to the FAO.

Nowadays, hazelnuts represent a relatively small, yet consistent, market portion in constant growth both in developed countries and in emerging economies, which is projected to grow by over 10% in the next 5 years. According to the FAO (2019), during 2019 more than 1 million tons of in-shell hazelnuts were harvested. The global production during 2017-2019 is visually summarized in Figure 1. Turkey is the main producer since it covers more than 67% of the global production, followed by Italy (≈12%), Azerbaijan (≈5%), and the USA (≈4%).

The Turkish production is mainly located in two areas along the Black Sea: the eastern area, which accounts for 60% and includes the provinces of Samsun, Ordu, Giresun, and Trabzon, and the western area, named Akçakoca, which accounts for the other 40% and includes the provinces of Sakarya, Zonguldak, Bolu, and Düzce. Turkish hazelnuts are usually supplied as a regional blend (*e.g.*, Akçakoca blend and Giresun blend), and depending on the area of interest, different cultivars are more abundant: in the eastern area (Giresun/Ordu) the most prominent cultivars are the Tombul, Çakildak, Mincane, and Palaz, while in the western area (Akçakoca) the leading cultivars are the Karafindik, Mincane, Çakildak, and Foşa (ISLAM, 2018). Among these cultivars, Tombul is the most abundant and has been described as the best Turkish cultivar in terms of overall kernel quality (Balik et al., 2018).

Italy is the second-largest producer with four main production areas: Campania (≈32%), where the cultivars are the Mortarella, San Giovanni, and Tonda di Giffoni; Piemonte (≈30%), with the cultivar Tonda Gentile Trilobata; Lazio (≈25%), with the cultivars Tonda Gentile Romana and Nocchione; and Sicilia (≈11%) where the cultivars are primarily used as fresh products and do not have an industrial interest.

In Azerbaijan, the main cultivar is the Ata-Baba which accounts for about 80% of the total hazelnut production and is concentrated in the Qabala and Qakh districts in the northwest of the country.

In the USA the production is concentrated in Oregon, on the pacific coast, which accounts for ≈99% of the total production. Barcelona is the main cultivar (≈60%), but many cultivars from artificial breeding are continuously developed to improve the resistance to *Phytocoptella avellanae*, a mite affecting local trees. Table 1 reports a summary of the main cultivars and harvest regions of the four largest producers.

**Table 1:** Summary of the four main hazelnut producers with the regions of interest and dominant cultivars for each region.

**Country**	**Tons (%)**	**Regions and Provinces**	**Cultivars**
Turkey	655,348 (67%)	Giresun, Ordu, Samsun and Trabzun – (60%)	Tombul, Çakildak, Mincane and Palaz
		Sakarya, Zonguldak, Bolu and Düzce – (40%)	Karafindik, Mincane, Çakildak and Foşa
Italy	120,837 (12%)	Campania (≈32%)	Mortarella, San Giovanni and Tonda Giffoni
		Piemonte (≈30%)	Tonda Gentile Trilobata
		Lazio (≈25%)	Tonda Gentile Romana, Nocchione and Tonda Giffoni
Azerbaijan	50,463 (5%)	Qabala and Qakh	Ata-Baba
USA	38,406 (4%)	Oregon	Barcelona

*Percentage values refer to the global production during the years 2017-2019*.

Table 2 summarizes the main characteristics of the most relevant hazelnut cultivars. It is important to highlight that industrially appealing hazelnut cultivar characteristics include thin shell, easy cuticle removal (high blanching rate), high shelling yield (kernel/nut ratio >45%), globular kernels, and a kernel caliber ideally around 13 mm (Caramiello et al., 2000).

**Table 2:** List of the most industrially used cultivars and their characteristics.

**Cultivars**	**Area of cultivation**	**Characteristics**
Ata-Baba	Azerbaijan	Small nut with plane-round shape, thin shell
Barcelona	Oregon (USA), Chile, France	Large nut with conical shape (RI = 0.92), 43% kernel/nut ratio, intermediate blanching rate, thick shell.
Çakildak	Ordu (TR)	Small nut with globular shape (RI = 1), 46% kernel/nut ratio, high blanching rate, thin shell.
Foşa	Trabzon (TR), Akçakoca (TR)	Small nut with globular shape (RI = 0.97), 50% kernel/nut ratio, high blanching rate, thin shell. Good flavor.
Karafindik	Akçakoca (TR)	Small nut with a long shape (RI = 0.83), 33.9% kernel/nut ratio, high blanching rate, thin shell
Mincane	Akçakoca (TR)	Small nut with a long shape (RI = 0.87), 47% kernel/nut ratio, high blanching rate, thin shell
Mortarella	Campania (IT)	Medium to small nut with short cylindrical shape (RI = 0.78), 45% kernel/nut ratio, high blanching rate, thin shell. Excellent flavor.
*Nocchione*	Lazio (IT)	Medium to large nut with globular shape (RI = 0.97), 38% kernel/nut ratio, intermediate blanching rate, thick shell.
Palaz	Ordu (TR)	Medium nut with globular shape (RI = 0.99), 47% kernel/nut ratio, very high blanching rate, thin shell. Good flavor.
San giovanni	Lazio (IT)	Medium nut with short cylindrical shape (RI = 0.76), 46% kernel/nut ratio, intermediate blanching rate, intermediate shell thickness.
Tombul	Turkey (All regions)	Small nut with conical shape (RI = 0.83), 50% kernel/nut ratio, very high blanching rate, thin shell. Excellent flavor.
Tonda giffoni	Campania (IT)	Medium nut with globular shape (RI = 1), 46% kernel/nut ratio, high blanching rate, thin shell. Excellent flavor. European labeling: PDO-IT-0573-AM01
Tonda gentile trilobata	Piemonte (IT)	Small to medium nut with flat shape (RI = 0.98), 45.5% kernel/nut ratio, high blanching rate, thin shell. Excellent flavor. European labeling: PGI-IT-0305-AM03
Tonda gentile romana	Lazio (IT)	Medium nut with globular shape (RI = 0.98), 45% kernel/nut ratio, intermediate blanching rate, thin shell. Very good aroma. European labeling: PDO-IT-0573-AM01

This review focuses on the chemistry of the volatilome of high-quality hazelnuts; in particular, it systematically presents information about the distribution of potent odorants and key-aroma compounds in raw and roasted hazelnuts and critically discusses them given the increasing market demand for high-quality products for the confectionery industry.

The impact of post-harvest practices, storage conditions, and roasting (i.e., the key-technological process) is examined for their impact on volatiles signatures and the development of positive and negative sensory attributes. The correlation between primary metabolites and key-odorants signatures, by the *aroma potential* concept (Cialiè Rosso et al., 2018, 2020, 2021), is also discussed as a new perspective for the application of modern *omics* strategies to hazelnut research (Miguel et al., 2011; Stilo et al., 2021a).

## Hazelnuts Composition and Its Correlation to Sensory Properties

A characteristic composition with a peculiar balance between *primary* and *specialized* (formerly referred to as *secondary*) metabolites is at the basis of the pleasant sensory profile and potential health benefits of hazelnuts. Taste-active components, interacting with chemoreceptors located in the oral cavity, trigger basic taste sensations (i.e., sweet, acid, bitter, salty, and umami); they are generally connoted by lower volatility accompanied by a high polarity and water solubility. In hazelnut, major taste active compounds are free amino acids, sugars, organic acids (i.e., primary metabolites), phenolic acids, and condensed tannins (i.e., specialized plant metabolites) (Alasalvar et al., 2010, 2012b).

Some of these non-volatile constituents belonging to the class of specialized metabolites (i.e., phenolic derivatives in aglycones or glycosides) can also trigger trigeminal sensations (i.e., chemestesis) while eliciting velvety and astringency sensations (Schieberle and Hofmann, 2011).

On the other hand, aroma-active components are characterized by low water solubility, medium-to-low polarity, and molecular weight below 300 Da. The interaction of odor-active volatiles with the array of Olfactory Receptors (ORs) in the olfactory epithelium activates a complex signals pattern, i.e., the Receptor Code (Firestein, 2001; Breer et al., 2006; Audouze et al., 2014; Dunkel et al., 2014). These olfactory stimuli activate the nervous system for cognitive mechanisms of learning and experience, and as the ultimate event the olfactory perception.

Aroma perception alone is responsible for up to 80% of the whole hedonic profile (Dunkel et al., 2014) of food. The synergy between taste and aroma perception, also referred to as flavor (Schieberle and Hofmann, 2011), is at the basis of positive consumer experience and of the hedonic quality of hazelnuts.

This review has hazelnut volatilome as a primary focus and, within the bulk of its detectable volatiles, those potent odorants capable of eliciting positive or negative sensations perceivable during hazelnut consumption.

The next section briefly summarizes the bulk composition of raw hazelnuts by presenting major primary and specialized metabolites classes with a focus on those components that are also potent odorant precursors.

### Primary Metabolites

Hazelnuts are an all-round source of nutrients; in this perspective, the lipid fraction is the most abundant, it accounts for 50–73% of the total composition (Köksal et al., 2006), followed by 10–22% of carbohydrates and 10–24% of proteins; moisture (≈5%) and ashes (≈3%) complete the profile.

### Lipids

The lipid fraction is characterized by a saponifiable portion further classified in a polar fraction that accounts for 1.2% of the total amount and is represented by phosphatidylcoline, phosphatidylethanolamine, and phosphatidylinositol (Alasalvar et al., 2003b), and an apolar fraction of about 98.8%, consisting of triacylglycerols of which the principal contributor is oleic acid (18:1ω9) with 77.5–82.95%, followed by linoleic acid (18:2ω6) that is responsible for the 7.55–13.69% and by palmitic acid (16:0) that constitutes the 4.85–5.79% of the total. A summary of the most abundant fatty acids present in 16 *varieties/cultivars* are illustrated in Table 3 (Köksal et al., 2006).

**Table 3:** Fatty acid composition of 16 hazelnut varieties, data expressed in g/100g.

**Variety**	**C16:0**	**C16:1**	**C18:0**	**C18:1**	**C18:2**	**C18:3**
Cavacava	5.87	0.22	2.37	78.8	12.7	0.069
Çakildak	4.89	0.32	2.15	80.7	11.9	0.059
Foşa	5.62	0.37	1.70	79.0	13.2	0.074
Incekara	5.67	0.32	1.76	79.5	12.7	0.073
Kalinkara	5.71	0.42	2.42	79.5	11.9	0.067
Kan	5.72	0.32	2.30	81.8	9.82	0.053
Karafindik	5.62	0.28	2.37	78.9	12.8	0.058
Kargalak	4.89	0.42	0.86	81.0	12.7	0.067
Kuş	5.69	< LOQ	0.87	79.9	13.5	0.076
Mincane	5.02	0.38	1.90	82.8	9.89	0.029
Palaz	4.87	0.34	2.13	77.6	15.0	0.076
Sivri	4.72	0.42	2.49	79.2	13.2	< LOQ
Tombul	5.17	0.48	1.75	77.8	14.8	0.054
Uzunmusa	5.70	0.46	1.41	78.8	13.6	0.069
Yassi Badem	4.87	0.28	1.43	81.1	12.2	0.046
Yuvarlak Badem	5.66	0.36	0.87	74.2	18.73	< LOQ

*Table adapted by Köksal et al. (2006)*.

The unsaponifiable fraction is mostly characterized by sterols, which differ in composition between cultivars and geographical origin, and decreases along with the shelf-life (Amaral et al., 2006; Ilyasoglu, 2015; Ghisoni et al., 2020). The three most abundant sterols are β-sitosterol, accounting for 85% of the total content, campesterol at 5–7%, and Δ5-avenasterol, which achieves on average 2–4% of the total composition (Ilyasoglu, 2015). The sterol fraction plays an important role in authentication and frauds counteraction, it is used to identify olive oils adulterated with hazelnut oils of lower quality (Parcerisa et al., 1999; Zabaras and Gordon, 2004), as it might be further used in the discrimination of different hazelnut cultivars (Ghisoni et al., 2020).

The lipid fraction is at the basis of major sensory defects developed during hazelnut storage (Kinderlerer and Johnson, 1992; Alasalvar et al., 2003b; Azarbad and Jeleń, 2015; Ghirardello et al., 2016; Cialiè Rosso et al., 2018); unsaturated fatty acids are prone to autoxidation forming hydroperoxides derivatives further degraded in secondary products with lower polarity, molecular weight and odor threshold (OT) (Belitz et al., 2009). Moreover, TAGs are hydrolyzed and free fatty acids (FFAs) are more easily oxidized by the enzymatic activity, mainly promoted by endogenous esterases and lipases (Cialiè Rosso et al., 2021).

### Proteins and Free Amino Acids

The hazelnut protein content is on average 18% in weight (Kamal-Eldin and Moreau, 2009). Proteins can be classified into two main groups: albumins and globulins with an aminoacidic composition where glutamic acid (2.84–3.71 g/100 g), arginine (1.87–2.21 g/100 g), and aspartic acid (1.33–1.68 g/100 g) are the more abundant reaching 30% of the total fraction (Durak et al., 1999; Köksal et al., 2006; Ramalhosa et al., 2011). Table 4 shows in more detail the free amino acid profiles of hazelnuts from different geographical areas.

**Table 4:** Amino acids composition (g/100g) of hazelnuts from different geographical origin.

**Amino acids**	**Tombul**	**Turkey^**a**^**	**USA^**b**^**	**New Zealand^**c**^**
Alanine	0.7	0.72	0.73	0.54
Arginine	2.16	2	2.21	1.87
Aspartic acid	1.52	1.49	1.68	1.33
Cysteine	0.46	Unidentifiable	0.28	0.27
Glutamic acid	3.13	2.84	3.71	2.86
Glycine	0.71	0.64	0.72	0.54
Histidine	0.45	0.42	0.43	0.32
Hydroxyproline	0.06	Unidentifiable	Unidentifiable	Unidentifiable
Isoleucine	0.58	0.56	0.55	0.47
Leucine	1.07	1.15	1.06	0.82
Lysine	0.41	0.45	0.42	0.42
Methionine	0.23	0.16	0.22	0.18
Phenylalanine	0.66	0.64	0.66	0.56
Proline	0.56	0.59	0.56	0.49
Serine	0.65	0.72	0.74	0.56
Threonine	0.53	0.46	0.5	0.39
Tryptophan	0.04	Unidentifiable	0.19	Unidentifiable
Tyrosine	0.53	0.47	0.36	0.4
Valine	0.71	0.66	0.7	0.6

*Table adapted from Alasalvar et al. (2003b) and Alasalvar and Shahidi (2008). “a” varieties Aci, Cavcava, Çakildak, Foşa, Incekara, Kalinkara, Kan, Karafindik, Kargalak, Kus„ Mincane, Palaz, Sivri, Tombul, Uzunmusa, Yassi Badem, and Yuvarlak Badem. “b” unknown varieties. “c”: varieties Whiteheart, Barcelona, Butler, Ennis, Tonda di Giffoni, and Campanica*.

Caligiani et al. (2014) mapped the primary metabolome of selected raw and roasted hazelnuts from Tonda Gentile Trilobata (Piemonte TGT, Italy), Tonda Giffoni (Lazio, Italy), and Turkish varieties (Caligiani et al., 2014) by nuclear magnetic resonance (NMR) spectroscopy. Tryptophan was the discriminant marker for the Turkish hazelnuts while higher concentrations of choline and acetic acid characterized TGT samples.

Besides their role as markers for authentication, amino acids are also non-volatile precursors of several potent odorants responsible for pleasant notes in roasted hazelnuts. In a recent study, Cialiè Rosso et al. (2020, 2021) investigated the amino acidic patterns in raw and roasted hazelnuts while observing linear correlations between aroma precursors in raw hazelnuts and potent odorants in lab-scale roasted hazelnuts.

### Carbohydrates

Carbohydrates can reach 26% of the total composition; they can be further divided into dietary fiber, sugars, and starch (Coelho et al., 2007; Alasalvar and Shahidi, 2008). In unroasted hazelnuts, the percentage of total non-resistant and resistant starch expressed as % on a dry basis (d.b.), is 2.71 ± 0.08, 1.190 ± 0.07, and 1.52 ± 0.01, respectively (Alasalvar and Shahidi, 2008).

Sugars reach 17% of the total composition; they include di-saccharides (sucrose, stachyose, raffinose), monosaccharides (glucose and fructose), and polyalcohols (myo-inositol). The total sugar content of hazelnut is, on average, around 3.58 g/100 g, and the sucrose achieves about 74% of the total (Alasalvar and Shahidi, 2008; Sciubba et al., 2014). Sugars contribute directly and indirectly to hazelnut sensorial profile: they are responsible for the sweet taste of raw nuts and represent fundamental precursors of aroma active compounds since they react within the Maillard reaction framework and degrade during roasting (Cristofori et al., 2008; Kiefl, 2013; Kiefl and Schieberle, 2013; Taş and Gökmen, 2018).

Bonvehí and Coll (1993) studied the carbohydrates fraction and its variations as a function of varieties/cultivar and harvest region; their findings indicated that starch and fiber were almost stable while soluble sugars were highly variable within varieties, with the mountain harvested varieties containing a higher sucrose amount.

### Specialized Metabolites

Phenolic derivatives are the most abundant compounds in hazelnut kernels amongst specialized metabolites (Shahidi et al., 2007; Alasalvar and Shahidi, 2008; Alasalvar and Bolling, 2015; Bottone et al., 2019). Among them, phenolic acids of the hydroxybenzoic acid series are represented by gallic acid, the most abundant, followed by *p-*hydroxybenzoic acid, salicylic acid, 4-hydroxysalicylic acid, vanillic acid, and syringic acid. Among the hydroxycinnamic derivatives, *o-* and *p-*coumaric acid, caffeic acid, ferulic and isoferulic acid, and sinapic acid were identified in hazelnut kernels (Yuan et al., 2018).

Polyphenols are instead represented by quercetin, myricetin, and rutin, present in kernels as aglycones and/or as *O-*glycosides (e.g., quercetin 3-rhamnoside, quercetin-3-glucoside, and myricetin 3-rhamnoside) (Bottone et al., 2019). Among flavonoids, catechin, epicatechin, and epigallocatechin were identified in kernels (Prosperini et al., 2009; Fanali et al., 2018) accompanied by a complex fraction of their polymers (i.e., proanthocyanidins and condensed tannins) with procyanidins A2, B1, and B2 as major congeners (Fanali et al., 2018; Bottone et al., 2019).

Phenol signatures were effectively exploited for cultivar discrimination by Ciarmiello et al. (2014) who analyzed 29 European cultivars and, based on the total polyphenolic content and the qualitative composition, defined some potential markers for quality control.

Recently, by untargeted metabolomic investigation based on liquid chromatography and high-resolution mass spectrometry (LC-ESI-qTOF MS), Ghisoni et al. (2020) profiled six hazelnut cultivars harvested in Chile, Georgia, Italy, and Turkey. More than 1,000 polyphenols and sterols were annotated and tracked among samples. Flavonoids (anthocyanins, flavanols, and flavonols), phenolic acids (mainly hydroxycinnamics) together with sterols (i.e., cholesterol, ergosterol, and stigmasterol derivatives) were defined as putative markers for geographical origin discrimination.

Phenols and polyphenols, also present in hazelnut perisperm cuticle, are responsible for the taste and chemesthetic attributes (bitterness, astringency, velvety sensations), and during roasting may form phenolic volatiles eliciting *smoky* and *phenolic* odors, the latter being distinctive of high-quality cultivars (Burdack-Freitag and Schieberle, 2012; Kiefl et al., 2013).

Another class of specialized metabolites is that of monoterpenoids: they are biosynthesized from C5 precursors (i.e., dimethyl allyl pyrophosphate and isopentenyl pyrophosphate) in plants and are present in raw and roasted hazelnut kernels as distinctive native signatures. The most reported are pinenes (α-pinene and β-pinene) (Alasalvar et al., 2003a; Cordero et al., 2010; Burdack-Freitag and Schieberle, 2012; Kiefl, 2013; Cialiè Rosso et al., 2018), linalool and limonene (Burdack-Freitag and Schieberle, 2012; Cialiè Rosso et al., 2018), δ-3-carene (Alasalvar et al., 2003a), and β-caryophyllene, a sesquiterpenoid detected in Turkish *Tombul* hazelnuts (Alasalvar et al., 2003a).

Recently, by applying comprehensive two-dimensional gas chromatography (GC×GC), which enables highly effective fingerprinting of volatiles in many foods, a detailed signature of terpenoids was delineated. It includes α-pinene, β-pinene, (*E*)-*p-*2-menthen-1-ol, camphene, δ-3-carene, α-thujene, γ-terpinene, sabinene, limonene, *cis-*sabinene hydrate, α-terpinolene, β-phellandrene, and *p-*cymene (Kiefl, 2013; Kiefl et al., 2013; Nicolotti et al., 2013b; Cialiè Rosso et al., 2018). In particular with specialized metabolites, α-damascone and (*E*)-β-damascenone, nor-isoprenoids formed by oxidative cleavage of carotenoids, are of relevance for hazelnut aroma (Burdack-Freitag and Schieberle, 2010).

## Hazelnut Volatilome

The *volatilome*, also referred to as *volatome* (Phillips et al., 2013; Broza et al., 2015), “*contains all of the volatile metabolites as well as other volatile organic and inorganic compounds that originate from an organism*” (Amann et al., 2014), super-organism, or ecosystem. In line with this definition, all volatile metabolites present in the volatilome belongs to the sample's metabolome, although in this complex fraction it could be present degradation components or exogenously formed compounds not generated by plant metabolic processes [e.g., environmental contaminants, compounds formed by bacteria and molds metabolic processes- *microbial cloud* (Meadow et al., 2015), etc.].

The volatilome is therefore a distinct entity from the metabolome, it gives access to a higher level of information about many biological phenomena related to plant and food quality through its multiple chemical dimensions (Giddings, 1995). The ultimate analytical solutions to investigate food volatilome are those adopting high-resolution separations (e.g., mono-dimensional gas chromatography−1DGC; heart-cut two-dimensional gas chromatography –H/C-2DGC; or comprehensive two-dimensional gas chromatography - GC×GC) combined to low-resolution or high-resolution mass spectrometry (MS). Insights on analytical strategies for comprehensive investigations of food volatiles are outside the scope of this review; however, for interested readers, here follow some reference papers of interest (Sides et al., 2000; Tranchida et al., 2013; Cordero et al., 2015, 2019; Franchina et al., 2016; Pedrotti et al., 2021; Stilo et al., 2021a).

The food volatilome is a complex mixture of volatiles belonging to several different chemical classes as a function of the main metabolisms and reactions contributing to its expression. Regarding hazelnuts, native volatiles are those formed along with the terpenoid biosynthesis, from isopentenyl pyrophosphate and dimethyl allyl pyrophosphate as precursors (Dewick, 1986). They are a direct expression of the plant phenotype although recent findings correlated their presence to bacteria and mold development during storage (Stilo et al., 2021b).

Other important volatiles present in the hazelnut volatilome are secondary products of lipid oxidation. They are degradation products (β-scission and hydroperoxide epi-dioxide decomposition) of fatty acids hydroperoxides formed by lipids autoxidation. Within this group, several low molecular weight carbonyl derivatives (linear saturated aldehydes, unsaturated aldehydes, methyl-ketones), hydrocarbons, alcohols, and short-chain fatty acids can be found (Kinderlerer and Johnson, 1992; Belitz et al., 2009). When post-harvest practices do not properly stabilize kernels before storage, fruit germination might occur as well as bacteria and molds could find optimal conditions to grow (water activity – *a*_*w*_, temperature, and substrates availability) increasing volatilome chemical complexity. As a consequence, primary and secondary alcohols, carboxylic acids from fermentation reactions (Cialiè Rosso et al., 2018), lactones by cyclization of hydroxyl substituted fatty acids, furans by glucose and reducing sugars degradation, and some aromatic derivatives (benzaldehyde, phenyl ethyl alcohol, phenyl ethyl acetaldehyde, alkylated phenol derivatives) by non-volatile precursors like amino acids and phenolic compounds can be found (Cialiè Rosso et al., 2018).

Industrial processing has its impact on primary food materials and is generally designed to obtain optimal hedonic properties including desirable flavor, texture, and color (Alasalvar et al., 2003a, 2006; Nicolotti et al., 2013a). Specifically, for hazelnuts, industrial roasting promotes the formation of a complex array of volatile compounds that concur with the volatilome complexity. Medium-polarity, low-molecular-weight technological markers belong to many different classes: alcohols, Strecker aldehydes, ketones, and di-carbonyls formed within the Maillard reaction framework (Cordero et al., 2008, 2010; Kiefl et al., 2013), acids, esters, lactones, sulfur derivatives, and alkylated heterocycles (furans, pyrazines, pyrroles, thiophenes, aromatic compounds, phenols, pyridines, thiazoles, oxazoles) (Saklar et al., 2001; Alasalvar et al., 2003a; Seyhan et al., 2007; Burdack-Freitag and Schieberle, 2010, 2012; Kiefl et al., 2012, 2013; Kiefl, 2013).

The next paragraphs report and discuss major findings of the hazelnut volatilome by focusing on specific functional variables with a strong correlation to sensory quality.

### Cultivar/Origin Signatures in the Hazelnut Volatilome

Alasalvar et al. (2004, 2012a) dedicated several research projects to delineate distinctive signatures of potent odorants in high-quality hazelnuts from Turkey. In a study on Giresun and Tombul hazelnuts (harvest year 2001), authors applied dynamic headspace analysis (DHA) combined with gas chromatography coupled with mass spectrometry (GC-MS) for high informative profiling of volatiles. A total of 79 volatile compounds were identified by matching linear retention index (*I*^*T*^) and EI-MS spectral signatures with authentic standards and/or data from commercial databases. A total of 39 compounds were detected in raw hazelnuts; of them, 32 were identified as ketones (10), aldehydes (8), alcohols (5), aromatic hydrocarbons (5), and furans (4). Some of these components, as potent odorants, were positively correlated with specific and peculiar odor qualities revealed by descriptive sensory analysis (DSA) run by a trained panel.

Of these compounds, within ketones, the most odor impacting was 5-methyl-(*E*)-2-hepten-4-one (i.e., filbertone) with a typical *nutty* and *hazelnut*-*like* aroma. (*E*)-3-penten-2-one was correlated to a *fruity* odor while 2,3-pentanedione to *sweet, buttery*, and *caramel*-*like* notes. In the aldehydes class, eight congeners were found in raw kernels, 2-methylpropanal, 2- and 3-methylbutanal were reported to be responsible for *fruity, malty, nutty*, and *chocolate*-*like* odors. In addition, linear saturated and monounsaturated aldehydes (*i.e.*, hexanal, heptanal, nonanal, (*E*)-2-hexanal, and (*E,E*)-2,4-hexadienal) were responsible for perceivable *green, fatty, sweet floral*, and *fruity* notes. They are strongly correlated to lipid autoxidation and have a role in the sensorial quality degradation of kernels along with shelf-life (Cialiè Rosso et al., 2018).

Five alcohols were detected by dynamic headspace (DHS-)GC-MS in raw hazelnuts: 3-methyl-1-butanol was correlated to *dark chocolate, pungent*, and *sweet* odors while 1-pentanol was correlated with *rancid, burnt, wine-like* notes, 1-hexanol typical of *green* and *fatty* smells, 1-octanol and 1-octen-3-ol, most probably formed by fatty acids hydroperoxide decomposition, were connoted by *musty* and *mushroom*-like odors.

Aromatic hydrocarbons, like toluene, 1,2,4-trimethylbenzene, and 1,2,3-trimethylbenzene were more abundant in roasted kernels although their presence was above the method limit of detection (LOD) for raw hazelnuts.

Raw hazelnut volatilome mapping greatly improved, in terms of the number of volatiles detected and the method's sensitivity, with the application of GC×GC-TOF MS combined with high concentration capacity (HCC) sampling (Bicchi et al., 2004). Cialiè Rosso et al. reported the reliable identification of 133 volatiles in raw hazelnuts from Ordu (Turkey), and Lazio (Tonda Gentile Romana, Italy); most of them were already cross-mapped in other cultivars/origins (e.g., Tonda Giffoni, Tonda Gentile Trilobata, Mortarella, Akçakoca, Giresun, Trabzon, and Chile) by GC×GC-qMS (Cordero et al., 2010; Nicolotti et al., 2013a). Table 5 reports a consensus list of characteristic volatiles together with their experimental *I*^*T*^ on polar columns, odor qualities, and OTs in oil or air.

**Table 5:** Volatilome composition detected in raw and roasted hazelnut samples.

**Compound**	**CAS**	**Odor quality**	**OT (μg/kg)**	**^**1**^D *I^***T***^* (polar)**	**Chemical class**	**Reference**
(E)-2-Decenal	3913-71-1	Fatty, tallowy, orange-like	101^∧^	1,629	Aldehydes	A, B
(E)-2-Heptenal	18829-55-5	Fatty, almond-like	14^×^	1,306	Aldehydes	A, B
(E)-2-Hexenal	6728-26-3	Green, fresh	0.42^×^	1,202	Aldehydes	A, C
(E)-2-Nonenal	18829-56-6	Fatty, cucumber-like	4.1^∧^	1,510	Aldehydes	A, B
(E)-2-Octenal	2548-87-0	Fatty, nutty	50^∧^	1,415	Aldehydes	A, B
(E)-2-Pentenal	1576-87-0	Pungent, apple-like	300^×^	1,024	Aldehydes	B
(E)-2-Undecenal	53448-07-0	Fruity, waxy	150,000^×^	1,747	Aldehydes	A
(E)-3-Hepten-2-one	1119-44-4	\	\	1,277	Ketones	A
(E)-3-Penten-2-one	3102-33-8	\	\	1,102	Ketones	B, C, D
(E)-Methyl-2-octenoate	7367-81-9	Sweet, fruity	\	1,386	Esters	A
(E,E)-2,4-Decadienal	25152-84-5	Fatty	166^*^	1,783	Aldehydes	E
(E,E)-2,4-Hexadienal	142-83-6	Green	270^×^	1,411	Aldehydes	C
(E,E)-2,4-Nonadienal	5910-87-2	Fatty, waxy	1.5^∧^	1,680	Aldehydes	E, A
(Z)-3-Penten-2-one	3102-32-7	\	\	1,001	Ketones	B
1(H)-Pyrrole-2-carbaldehyde	1003-29-8	Musty, beefy	65,000^×^^$^	2,024	Heterocycles	B, D
1,2,3-Trimethylbenzene	526-73-8	\	\	1,301	Aromatics	A, C
1,2,4-Trimethylbenzene	95-63-6	\	\	1,283	Aromatics	C
1,3-Dimethylbenzene	108-38-3	Plastic	1,000^×^£	1,122	Aromatics	A
1-Butanol	71-36-3	Fruity	3,112^×^	1,089	Alcohols	B
1-Cyclopentyl ethanone	6004-60-0	\	\	1,203	Ketones	C
1-Decene	872-05-9	\	\	1,033	Alkanes	A
1-Dodecanol	112-53-8	Waxy, soapy	1,200^×^£	1,960	Alcohols	A
1-Heptanol	111-70-6	Cucumber, citrus-like	20,000^×^	1,438	Alcohols	A, B, C
1-Hepten-3-ol	4938-52-7	Green, oily, tomato-like	3000^×^	1,367	Alcohols	B
1-Heptene	592-76-7	\	\	828	Alkanes	A
1-Hexanol	111-27-3	Green, flowery	400^×^	1,338	Alcohols	A, B, C
1-Hydroxy-2-propanone	116-09-6	Pungent, sweet, caramellic	10,000^×^^$^	1,249	Ketones	B, D
1-Methylpyrrole	96-54-8	Smoky, woody, herbal	\	1,560	Heterocycles	D
1-Nonanol	143-08-8	Floral, waxy	280^◦^	1,652	Alcohols	A, B
1-Nonene	124-11-8	\	26,000^×^	930	Alkanes	A
1-Octanol	111-87-5	Chemical, metal, burnt	900^×^	1,550	Alcohols	A, B, C
1-Octen-3-ol	3391-86-4	Mushroom-like	1^×^	1,425	Alcohols	A, B
1-Octen-3-one	4312-99-6	Mushroom-like	10^×^	1,289	Ketones	E, A, C
1-Octene	111-66-0	Gasoline	2,000^×^	851	Alkanes	A
1-Pentanol	71-41-0	Balsamic	470^×^	1,239	Alcohols	A, B, C
1-Tetradecanol	112-72-1	Waxy, fruity	5,000^×^^$^	2,183	Alcohols	A
2-(1-Pyrrolyl)ethanol	22186-60-3	\	\	1,949	Alcohols	B
2-(2-Ethoxyethoxy)-ethanol	111-90-0	Slightly ethereal	6,000^×^£	1,572	Alcohols	A
2(3H)-Furanone	20825-71-2	\	\	1,760	Lactones	B
2,2-Dimethyl-3-hexanone	5405-79-8	\	\	1,070	Ketones	B
2,3,5-Trimethylfuran	10504-04-8	\	\	1,037	Heterocycles	B, C, D
2,3,5-Trimethylnaphthalene	2245-38-7	Earthy	\	1,562	Aromatics	C
2,3,5-Trimethylpyrazine	14667-55-1	Earthy	290^×^	1,376	Pyrazines	E, C, B, D
2,3-Butanediol	513-85-9	Fruity, onion-like	20,000^×^	1,593	Alcohols	D
2,3-Butanedione	431-03-8	Buttery	9.2^∧^	983	Ketones	E, B
2,3-Diethyl-5-methylpyrazine	18138-04-0	Earthy, roasty	0.5^*^	1,470	Pyrazines	E
2,3-Dimethylpyrazine	5910-89-4	Nutty, cocoa-like	880^×^£	1,338	Pyrazines	D
2,3-Pentanedione	600-14-6	Buttery	0.3^∧^	1,049	Ketones	E, C, B, D
2,4-Dimethyl-3-pentanol	600-36-2	\	\	1,409	Alcohols	A, B
2,5-Dimethylfuran	625-86-5	Ethereal, chemical	100,000^×^£	940	Aldehydes	A, B, C, D
2,5-Dimethylpyrazine	123-32-0	Nutty, cocoa-like	2,600^*^	1,317	Pyrazines	C, D
2,6-Dimethylpyrazine	108-50-9	Nutty, cocoa-like	8,000^×^	1,323	Pyrazines	C, D
2-Acetyl-1-pyrroline	85213-22-5	Popcorn-like, roasty	0.092^*^	1,310	Heterocycles	E, B
2-Acetyl-1,4,5,6-tetrahydropyridine	25343-57-1	Popcorn-like, roasty	1.2^*^	1,538	Heterocycles	E
2-Acetyl-3,4,5,6-tetrahydropyridine	27300-27-2	Popcorn-like, roasty	1.2^*^	1,538	Heterocycles	E
2-Acetylfuran	1192-62-7	Sweet, balsamic, almond-like	15,000^×^^$^	1,488	Heterocycles	B
2-Acetylpyridine	1122-62-9	Popcorn-like, earthy	500^*^	1,564	Heterocycles	E
2-Butanone	78-93-3	Etheric	40,000^×^	952	Ketones	A, B, D
2-Butenal	4170-30-3	\	\	1,020	Aldehydes	B, D
2-Butylfuran	4466-24-4	Fruity, spicy	10,000^×^	1,110	Heterocycles	A
2-Cyclopenten-1,4-dione	930-60-9	\	\	1,581	Ketones	B
2-Decanone	693-54-9	Floral, fermented	60^×^£	1,471	Ketones	A, B
2-Ethyl-1-hexanol	104-76-7	Citrus, fresh, floral	270^×^£	1,471	Alcohols	B
2-Ethyl-2-hexenal	645-62-5	\	\	1,255	Aldehydes	B
2-Ethyl-3-methylpyrazine	15707-23-0	Fruity, sweet	130^×^£	1,399	Pyrazines	C
2-Ethyl-5-methylfuran	1703-52-2	Terpenic, sweet	\	1,021	Heterocycles	A, B, C
2-Ethyl-5-methylpyrazine	13360-64-0	Fruity, sweet	320^×^	1,373	Pyrazines	B, C, D
2-Ethyl-6-methylpyrazine	13925-03-6	Fruity, sweet	40^×^^$^	1,366	Pyrazines	B, C, D
2-Ethylhexanal	123-05-7	\	\	1,134	Aldehydes	B
2-Ethylpyrazine	13925-00-3	Nutty, musty	17,000^*^	1,305	Pyrazines	B, C, D
2-Furfuryl mercaptan	98-02-2	Coffee-like, sulfury	0.37^*^	1,421	Heterocycles	E
2-Heptanol	543-49-7	Citrus, fruity	263^×^	1,297	Alcohols	A, B
2-Heptanone	110-43-0	Soapy, fruity	300^×^	1,168	Ketones	A, B, C
2-Heptylfuran	3777-71-7	Green, fatty	\	1,421	Heterocycles	A
2-Hexanol	626-93-7	Winey, chemical	50,000^×^£	1,195	Alcohols	A
2-Hexanone	591-78-6	Fruity, fungal	98^×^£	1,082	Ketones	B
2-Hexylfuran	3777-70-6	\	\	1,318	Heterocycles	A
2-Isopropyl-3-methoxypyrazine	25773-40-4	Pea-like, green pepper	0.05^∧^	1,414	Pyrazines	E
2-Methoxyphenol	90-05-1	Smoky, phenolic	15^*^	1,839	Alcohols	E
2-Methyl-(E)-2-butenal	1115-11-3	Green, fruity	500^×^^$^	1,094	Aldehydes	C, D
2-Methyl-1-butanol	137-32-6	Roasted, winey	480^×^	1,178	Alcohols	A
2-Methyl-1H-pyrrole	636-41-9	\	\	1,554	Heterocycles	B, C
2-Methyl-1-propanol	78-83-1	Solvent-like	660^×^£	1,060	Alcohols	A, B
2-Methylbutanal	96-17-3	Malty	9.2^∧^	964	Aldehydes	E, A, B, D
2-Methylbutyl propanoate	2438-20-2	Sweet, fruity, ethereal	28^×^^$^	1,095	Esters	B
2-Methylbutyric acid	116-53-0	Sweaty	22^*^	1,667	Carboxylic acids	E
2-Methylfuran	534-22-5	Ethereal	27,000^×^	879	Heterocycles	D
2-Methylfuran-3-one	41763-99-9	\	\	1,371	Heterocycles	B
2-Methylpropanal	78-84-2	Green, pungent	430^×^	807	Aldehydes	B, C, D
2-Methylpyrazine	109-08-0	Popcorn-like	27,000^*^	1,262	Pyrazines	C, D
2-Nonanol	628-99-9	Waxy	58^×^^$^	1,491	Alcohols	A
2-Nonanone	821-55-6	Fruity, soapy	100^×^	1,366	Ketones	A, B
2-Octanol	123-96-6	Mushroom, fat	100^◦^	1,460	Alcohols	A
2-Octanone	111-13-7	Earthy, dairy	510^×^	1,252	Ketones	A, B
2-Octene	111-67-1	\	\	847	Alkanes	A
2-Pentanol	6032-29-7	Seedy, sharp	470^×^	1,101	Alcohols	A, C
2-Pentanone	107-87-9	Fruity	350^×^£	958	Ketones	A, B, C, D
2-Pentylfuran	3777-69-3	Buttery, green bean-like	100^×^	1,221	Heterocycles	A, B, C, D
2-Phenoxyethanol	122-99-6	Floral, rose	\	2,143	Alcohols	A
2-Phenylethyl Alcohol	60-12-8	Floral, sweet	2,000^×^	1,912	Alcohols	A
2-Propionyl-1-pyrroline	133447-37-7	Popcorn-like, roasty	0.1^*^	1,405	Heterocycles	E
2-Pyrrolidinone	616-45-5	\	\	2,042	Heterocycles	B
2-Vinyl-5-methylfuran	10504-13-9	\	\	1,160	Heterocycles	B
3,5-Dimethyl-2-ethylpyrazine	13925-07-0	Earthy	3.4^*^	1,440	Pyrazines	E, B, C
3,5-Dimethyl-4-heptanone	19549-84-9	\	\	1,145	Ketones	B
3-Butenoic acid	625-38-7	\	\	1,627	Carboxylic acids	C
3-Ethyl-2,5-dimethylpyrazine	13360-65-1	Burnt, roasted	166^*^	1,431	Pyrazines	B, C, D
3-Furanmethanol	4412-91-3	\	\	1,655	Alcohols	C
3-Heptanol	589-82-2	Citrusy	240^×^^$^	1,266	Alcohols	A
3-Heptanone	106-35-4	Green, ketonic	80^×^^$^	1,129	Ketones	A
3-Hydroxy-2-butanone	513-86-0	Buttery	14^×^^$^	1,234	Ketones	B, D
3-Mercapto-3-methyl-1-butanol	34300-94-2	Meaty	4^×^£	1,643	Alcohols	E
3-Methyl-1-butanol	123-51-3	Whiskey, malty, burnt	100^×^	1,174	Alcohols	B, C
3-Methyl-1H-pyrrole	616-43-3	\	\	1,556	Heterocycles	B
3-Methyl-2-butanol	598-75-4	Musty, alcoholic	410^×^^$^	1,117	Alcohols	D
3-Methyl-2-cyclohexen-1-one	1193-18-6	Nutty, caramellic, phenolic	\	1,593	Ketones	B, C
3-Methyl-2-pentanone	565-61-7	\	\	1,013	Ketones	B, C, D
3-Methyl-3-pentanol	77-74-7	Fruity, green	2,500^×^^$^	1,523	Alcohols	B
3-Methyl-4-heptanol	1838-73-9	\	\	1,326	Alcohols	A, B
3-Methyl-4-heptanone	15726-15-5	Fruity, nutty	0.86^*^	1,119	Ketones	E, A, B
3-Methylbutanal	590-86-3	Malty	5^∧^	937	Aldehydes	E, A, C, B, D
3-Methylbutyric acid	503-74-2	Sweaty	320^×^	1,667	Carboxylic acids	E
3-Methylpyridine	108-99-6	Green, earthy, nutty	\	1,214	Heterocycles	D
3-(methylthio) propionaldehyde	3268-49-3	Musty, earthy	0.18^*^	1,443	Aldehydes	E
3-Methylundecane	1002-43-3	\	\	1,167	Alkanes	A
3-Nonen-2-one	14309-57-0	Fruity, fatty	30^×^£	1,482	Ketones	A
3-Octanone	106-68-3	Herbal, mushroom	1.3^×^£	1,240	Ketones	A
3-Octen-2-one	1669-44-9	Earthy, creamy	250^×^	1,382	Ketones	A
3-Penten-2-one	625-33-2	Musty, fishy, phenolic	1,200^×^^$^	1,104	Ketones	A
3-Pyrroline	109-96-6	\	\	1,395	Heterocycles	C
4-Ethenyl-2-methoxyphenol	7786-61-0	Clove-like, smoky	50^*^	2,222	Alcohols	E
4-Heptanol	589-55-9	Alcoholic	39,400^×^£	1,255	Alcohols	A
4-Heptanone	123-19-3	Fruity, cheesy	8.2^×^^$^	1,106	Ketones	A, B
4-Hydroxy-2,5-dimethyl-3(2H)-furanone	3658-77-3	Caramel-like, sweet	23^*^	2,053	Lactones	E, B
4-Hydroxy-3-methoxybenzaldehyde	121-33-5	Vanillic, sweet	181^*^	2,591	Aldehydes	E
4-Hydroxybutyric acid	591-81-1	Creamy, milky	\	1,604	Carboxilic acid	A
4-Methoxybenzaldehyde	123-11-5	Anise-like	120^*^	2,017	Aldehydes	E
4-Methylbenzaldehyde	104-87-0	Fruity, cherry	1.2^×^£	1,642	Aldehydes	C
4-Octanol	589-62-8	\	\	1,361	Alcohols	A
5-Methyl-(E)-2-hepten-4-one	102322-83-8	Nutty, fruity	3.8^*^	1,268	Ketones	E, A, C, B, D
5-Methyl-(Z)-2-hepten-4-one	134357-02-1	Nutty, fruity		1,183	Ketones	E, D
5-Methyl-2-furanmethanol	3857-25-8	\	\	1,725	Heterocycles	B
5-Methyl-2-heptanone	18217-12-4	\	\	1,247	Ketones	A, B, C
5-Methyl-3,4-heptanedione	13678-56-3	Fruity	\	1,159	Ketones	B
5-Methyl-5-hexen-2-one	3240-09-3	Fatty, green	\	1,300	Ketones	C
5-Methylfurfural	620-02-0	Almond-like, caramel-like, burnt sugar	1110^×^^$^	1,569	Heterocycles	B, D
6-Methyl-5-hepten-2-one	110-93-0	Citrus, green, musty	1,000^×^	1,334	Ketones	C
6-Undecanone	927-49-1	\	\	1,506	Ketones	A
Acetaldehyde	75-07-0	Pungent, fruity	0.22^×^	768	Aldehydes	A, B
Acetic acid	64-19-7	Sour	114^∧^	1,442	Carboxylic acids	E, A, C, B, D
Acetone	67-64-1	Solvent-like	460^◦^	831	Ketones	A, B
Acetonitrile	75-05-08	\	\	973	Solvent	A, B
Acetylpyrrole	1072-83-9	Nutty, anisic, sweet	2,000^×^£	1,976	Heterocycles	B
Benzaldehyde	100-52-7	Almond, fruity	60^×^	1,495	Aldehydes	A, D
Benzyl alcohol	100-51-6	Floral, fruity	2,546^×^^$^	1,880	Alcohols	A, B
Butanal	123-72-8	Green, fruity, pungent	150^×^	868	Aldehydes	A, B
Butanoic acid	107-92-6	Sweaty, rancid	135^×^	1,611	Carboxilic acid	A, B
Butyl benzoate	136-60-7	Balsamic, fruity	\	1,874	Esters	A
Butyl Butanoate	109-21-7	Fruity	28^×^	1,197	Esters	A, B
Chloroform	67-66-3	\	\	1,003	Solvent	A
Decanal	112-31-2	Orange skin-like, flowery	75^×^	1,475	Aldehydes	A, B
Decane	124-18-5	\	\	1,000	Alkanes	A
Decanoic acid	334-48-5	Fatty, soapy	230,000^×^	2,265	Carboxilic acid	A, B
Dichloromethane	75-09-2	\	\	918	Solvent	A
Dimethyl trisulfide	3658-80-8	Sulfury	2.3^∧^	1,352	Sulfides	E
Dodecane	112-40-3	\	\	1,198	Alkanes	A
Ethyl acetate	141-78-6	Solvent-like, fruity	10,000^×^	949	Esters	A, B, D
Ethyl benzene	100-41-4	\	\	1,107	Aromatics	A
Ethyl benzoate	93-89-0	Minty, sweet	0.6^×^£	1,658	Esters	A
Ethyl octanoate	106-32-1	Waxy, sweet	10^×^£	1,419	Esters	A
Furfural	98-01-1	Sweet	82,000^*^	1,438	Aldehydes	B, C, D
Furfuryl alcohol	98-00-0	Burnt	32,000^×^£	1,656	Alcohols	B, D
Heptanal	111-71-7	Fatty	50^×^	1,173	Aldehydes	A, B, C
Heptane	142-82-5	\	\	700	Alkanes	A, B, D
Heptanoic acid	111-14-8	Cheesy, fatty	100^◦^	1,950	Carboxilic acid	A, B
Heptyl acetate	112-06-1	Green, fresh	6,300^×^	1,358	Esters	A
Heptyl formate	112-23-2	Green, fatty	\	1,306	Esters	A
Heptyl heptanoate	624-09-9	Grassy, green	\	1,587	Esters	A
Hexanal	66-25-1	Green, leaf-like	276^*^	1,070	Aldehydes	A, B, C, D
Hexane	110-54-3	\	\	600	Alkanes	A, B, D
Hexanoic acid	142-62-1	Fatty, cheesy	700^◦^	1,854	Carboxilic acid	A, B
Hexyl acetate	142-92-7	Fruity, green	700^×^	1,251	Esters	A
Hexyl formate	629-33-4	Fruity, green	\	1,199	Esters	A
Limonene	138-86-3	Citrus-like	14,700^×^	1,145	Terpenoids	A, B, D
Menthol	2216-51-5	Minty	180,000^×^	1,623	Terpenoids	A
Methyl dihydro jasmonate	24851-98-7	Floral, sweet	\	2,276	Esters	A
Methyl pyruvate	600-22-6	\	\	1,182	Esters	B
n-Butyl ether	142-96-1	Ethereal	1300^×^	951	Ethers	A, B
Nonanal	124-19-6	Tallowy, fruity	610^×^	1,382	Aldehydes	A, B, C
Nonane	111-84-2	Gasoline	2,150,000^×^	896	Alkanes	A
Nonanoic acid	112-05-0	Waxy, cheesy	2400^×^	2,171	Carboxilic acid	A, B
Octanal	124-13-0	Fatty, green	51^∧^	1,264	Aldehydes	A, B
Octane	111-65-9	Gasoline	1,340,000^×^	800	Alkanes	A, B, D
Octanoic acid	124-07-2	Fatty, soapy	3,000^◦^	2,070	Carboxilic acid	A, B
Octyl acetate	112-14-1	Floral, waxy	£140^×^	1,451	Esters	A
Octyl ether	629-82-3	\	\	2,281	Ethers	A
o-Xylene	95-47-6	Geranium	1,600^×^£	1,188	Aromatics	C
p-Cymene	99-87-6	Fresh, citrus, terpenic	18,000^×^	1,218	Terpenoids	B
Pentanal	110-62-3	Pungent, almond-like	150^×^	957	Aldehydes	A, B
Pentanoic acid	109-52-4	Sweaty	400^×^	1,711	Carboxilic acid	A, B
Pentyl acetate	628-63-7	Fruity, ethereal	780^×^	1,151	Esters	A
Pentyl formate	638-49-3	Fruity, sweet, ethereal	\	1,100	Esters	A
Pentyl hexanoate	540-07-8	Fruity, sweet	\	1,496	Esters	A
Pentyl octanoate	638-25-5	Earthy, sweet	\	1,690	Esters	A
Phenylacetaldehyde	122-78-1	Honey, flowery	25^*^	1,634	Aldehydes	E, B
Phtalide	87-41-2	Sweet, coumarinic	\	2,317	Heterocycles	B
Propanal	123-38-6	Green, acetaldehyde-like	9.4^×^	820	Aldehydes	A
p-Xylene	106-42-3	Plastic	250^×^£	1,137	Aromatics	C
Pyranone	28564-83-2	\	\	2,236	Heterocycles	B
Pyrazine	290-37-9	Nutty, sweet	\	1,204	Pyrazines	D
Pyridine	110-86-1	Sour, fishy	920^×^	1,134	Heterocycles	B, D
Pyrrole	109-97-7	Nutty, sweet, ethereal	\	1,513	Heterocycles	B, C, D
Sabinene	3387-41-5	Terpenic	2,000^×^£	1,072	Terpenoids	B
Sabinene hydrate	546-79-2	Terpenic	10,000^×^£	1,540	Terpenoids	B
Styrene	100-42-5	Balsamic, almond	3,100^×^	1,245	Aromatics	A
Terpinen-4-ol	562-74-3	Spicy, cooling	590^×^£	1,553	Terpenoids	A
Tetradecane	629-59-4	Mild, waxy	13,000,000^×^	1,403	Alkanes	A
Tetrahydrofuran	109-99-9	\	\	859	Heterocycles	A
Toluene	108-88-3	Sweet	330^×^	1,038	Aromatics	A, B, C
trans-4,5-epoxy-(E)-2-Decenal	134454-31-2	Metallic	25^×^	1,993	Aldehydes	E
α-Pinene	7785-26-4	Herbal, woody	274^×^	1,012	Terpenoids	A, C
β-Caryophyllene	87-44-5	Sweet, woody	11,000^×^£	1,597	Terpenoids	C
γ-Butyrolactone	96-48-0	Creamy, oily, fatty	1,000^×^^$^	1,625	Lactones	B, D
γ-Heptalactone	105-21-5	Sweet, coconut, lactonic	3,400^◦^	1,806	Lactones	A, B
γ-Hexalactone	695-06-7	Sweet, creamy, lactonic	8,000^◦^	1,662	Lactones	A, B
γ-Nonalactone	104-61-0	Coconut	2,400^◦^	2,038	Lactones	A, B
γ-Octalactone	104-50-7	Sweet, coconut, lactonic	3,500^◦^	1,924	Lactones	A, B
γ-Pentalactone	129-69-8	Herbal, tonka	\	1,561	Lactones	A
γ-Terpinene	99-85-4	Terpenic, lime	55,000^×^£	1,241	Terpenoids	A, B
Δ-3-Carene	13466-78-9	Citrus, herbal	9,300^×^£	1,138	Terpenoids	A, C
δ-Heptalactone	3301-90-4	Coconut, creamy	\	1,879	Lactones	A

*Samples include fresh and spoiled samples from varieties Tonda Gentile Trilobata, Tonda Gentile Romana, Tonda Giffoni, Akçakoca, and Giresun blend. Experimental ^1^D retention index (I^T^) on DB-WAX or equivalent, odor threshold (OT) in oil or air, and odor quality included when available. Consensus table from references Alasalvar et al. (2003a); Nicolotti et al. (2013a); Cialiè Rosso et al. (2018), and Stilo et al. (2021b). (a) compound and experimental I^T^ from Stilo et al. (2021b); (b) compound and experimental I^T^ from Cialiè Rosso et al. (2018); (c) compound and experimental I^T^ from Alasalvar et al. (2003a); (d) compound and experimental I^T^ from Nicolotti et al. (2013a); (e) compound and experimental i^t^ from Kiefl and Schieberle (2013)*.

* *OT according to Kiefl and Schieberle (2013)*;

∧ *OT according to Burdack-Freitag and Schieberle (2012)*;

◦ *OT according to Stilo et al. (2021b)*;

× *OT according to Van Gemert (2003)*;

£ *OT reported in air (μg/m^3^) when OT in oil not available*;

$ *OT reported in water when OT in oil and air not available*.

### Effect of Post-harvest and Storage on Volatilome Signatures

The evolution of raw hazelnut volatilome along shelf-life was explored by Cialiè Rosso et al. (2018) in a study on commercial samples of Tonda Gentile Romana and on Ordu hazelnuts harvested in 2014. Samples were subjected to traditional sun-drying (D1 - ≈30/35°C) or artificial drying (D2) at low temperatures (≈18/20°C) in industrial plants. To study the effect of storage conditions, 5° and 18°C ± 0.1 were tested in combination with atmosphere composition as *regular* (NA: 78% N_2_-21% O_2_) or *modified* (MA 99% N_2_-1% O_2_).

Volatiles and potent odorants were sampled by headspace solid-phase microextraction (HS-SPME) and analyzed by GC×GC-TOF MS equipped with a thermal modulator. Analytical conditions enabled a suitable sensitivity by including in the fingerprinting/profiling process potent odorants and several key-aroma compounds (Burdack-Freitag and Schieberle, 2012; Kiefl et al., 2013).

The pattern of 133 known analytes, identified by matching EI-MS fragmentation patterns with those collected in commercial and in-house databases and *I*^*T*^ calculated on the ^1^D (±15 units of tolerance), was explored by multivariate statistics to highlight relevant features (i.e., components) with meaningful variations along storage time and as a function of storage conditions.

Explorative Principal Component Analysis (PCA) on analytes' response data indicated a natural conformation of sample groups according to cultivar/geographical origin, followed by the impact of a secondary variable, post-harvest drying conditions. Supervised univariate analysis by Fisher ratio (F), highlighted as relevant variables for post-harvest a series of linear and branched alcohols (2-heptanol, 2-methyl-1-propanol, 3-methyl-1-butanol, 2-ethyl-1-hexanol, benzyl alcohol), several esters (ethyl acetate, butyl butanoate, 2-methyl-butyl propanoate), and acetic acid. Most of them were already associated with nut ripening or fermentation (Zhou et al., 2013). Of interest, 3-methyl-1-butanol (*i.e*., isoamyl alcohol), a well-known fermentation product in must and wines, is formed from L-leucine. 2-methyl-1-propanol has instead L-valine as a precursor, while 2-heptanol is formed in tomatoes during ripening by β-ketoacids hydrolysis and subsequent decarboxylation (Fridman, 2005), and 2-ethyl-1-hexanol has been found in fermented soybean (Han et al., 2001).

By observing the evolution of 37 potent odorants within the detectable volatilome, Cialiè Rosso et al. (2018) confirmed the dominant role of drying conditions above cultivar/origin. Moreover, most potent odorants, with OTs up to 2,500 μg/L, were closely correlated (*r* > 0.800) to storage time. Of them, 1-heptanol (*green, chemical*), 2-octanol (*metal, burnt*), 1-octen-3-ol (*mushroom*), (E)-2-heptenal (*fatty, almond*), hexanal (*leaf*-*like, green*), heptanal (*fatty*), octanal (*fatty*), and nonanal (*tallowy, fruity*) are of great interest, since they might impart unpleasant notes in hazelnuts stored within 12 months. Some of the selected potent odorants showed increasing trends over time, achieving their maximum abundance at 12 months of storage. Those correlated to lipid oxidation, i.e., degradation products of fatty acids hydroperoxides (i.e., hexanal, octanal and (*E*)-2-heptanal) eliciting *fatty* and *green*-*leafy* notes (Pastorelli et al., 2007; Ghirardello et al., 2013), had a marked increase over time with higher relative ratios in samples subjected to sun drying. For those samples dried at lower temperatures in industrial plants, i.e., Tonda Gentile Romana, limited oxidation was detected, with amounts of hexanal and octanal at 2.6 and 2.8 times lower compared to standard drying conditions.

Moreover, 2-octanol and 1-octen-3-ol, formed by linoleic acid hydroperoxides cleavage promoted by fungal lipoxygenase/hydroperoxide lyase enzymes (Hung et al., 2014), are likely responsible for the *metallic* and *mushroom*-like notes. Their relative abundance was higher in Ordu samples accompanied by a marked increase with storage conducted in less protective conditions (18°C and NA 78% N_2_-21% O_2_). On the other hand, the same analytes were below method LOD in Tonda Gentile Romana hazelnuts dried at low temperatures.

Results on oxidative stability/instability are likely correlated to hazelnuts fatty acids profiles reported in several studies (Kinderlerer and Johnson, 1992; ÖZDEMIR, 1998; Özdemir et al., 2001; Koyuncu et al., 2005; Locatelli et al., 2015; Ghirardello et al., 2016; Belviso et al., 2017; Memoli et al., 2017; Momchilova et al., 2017; Turan, 2018; Pedrotti et al., 2021), where storage and processing were investigated for their impact on the fatty fraction.

However, recent findings on the evolution of FFAs along with shelf-life (Cialiè Rosso et al., 2021) suggest that post-harvest has a decisive impact on esterases/lipases activity; FFAs are more prone to oxidation than those esterified in TAGs, registering 10 times faster oxidation kinetics (Frega et al., 1999).

Potent odorants profiling indicated the decisive effect of post-harvest drying in preserving hazelnut quality and oxidation status; moreover, the marked development of potent odorants with unpleasant notes, formed by autoxidation of essential fatty acids, interestingly evokes the hypothesis that key flavor-related volatiles in vegetable food are generated from essential nutrients and health-promoting components (e.g., amino acids, fatty acids, and carotenoids), while informing the actual nutritional value of the product (Goff and Klee, 2006).

### Hazelnut Spoilage Volatiles Signatures

Hazelnuts quality might be degraded during growing, harvesting, and storage (Giraudo et al., 2018), resulting in some physical and sensorial defects. The industry implements several quality control procedures for incoming batches to check for physical damage: insect-damaged, rotten, twin, and yellowed hazelnut kernels (Mehlenbacher et al., 1993; Caligiani et al., 2014; Belviso et al., 2017; Göncüoglu Taş and Gökmen, 2017; Yuan et al., 2018). Visual inspection by trained staff on representative samples is, nowadays, the quality control procedure of choice in the hazelnut value chain. This approach, highly time-consuming, might be strongly influenced by the level of experience and sensibility of the operator (Giraudo et al., 2018).

Complex carbohydrates undergo enzymatic hydrolysis when attacked by worms, bacteria, or molds, to provide them an available source of metabolic energy. The autocatalytic oxidation of unsaturated fatty acids and the hydrolysis of lipids in free fatty acids are also triggered. These reactions result in a negatively altered aroma and taste, *i.e*., the *rotten* defect of the fruits (Giraudo et al., 2018). Physical damage, such as dark kernels with white/brown spots, arises from insect bites that transfer saliva enzymes to the nut (e.g., proteinases, esterases, lipases, and amylases). Furthermore, damaged fruit can be more easily attacked by *Aspergillus* and *Penicillium* species, the most common fungi found after rainy seasons (Pscheidt et al., 2019), whose metabolic activity might negatively impact the sensory properties of the nut.

Amrein et al. (2010, 2014) identified prenyl ethyl ether (PRE) as the cause of a *solvent* off-note in ground-haversted hazelnuts. The authors applied a sensory-oriented strategy based on olfactometry coupled to GC (i.e., GC-O), followed by odor value calculation and spiking experiments.

Solid-phase microextraction (SPME) and simultaneous distillation–extraction (SDE) were adopted to extract and isolate volatiles from hazelnut batches showing the *solvent* off-note. Based on GC-O, performed by a trained panel, an odor active region showing a *metallic solvent*-*like* aroma impression was identified. The presence of prenyl ethyl ether was confirmed by GC-MS and retention data.

Quantification results on a series of major volatiles referred that linear saturated aldehydes (hexanal, octanal, and nonanal), reported in many studies as responsible for *rancid* off-notes, did not show meaningful differences between defective and control samples. Interestingly, in defective samples that reported higher amounts of prenyl ethyl ether, several terpenes (myrcene, limonene, and valencene) were also present in high concentrations.

The authors went ahead to find the possible formation pathway for prenyl ethyl ether. Prenyl alcohols can be formed by *Aspergillus, Rhizopus, Penicillium, Eurotium, Mucor*, and *Fusarium*, therefore model experiments with contamination of hazelnuts with the abovementioned molds were conducted, unfortunately without success. To date, it is assumed that this *solvent metallic* component might be formed as a consequence of mold contamination in presence of unknown co-factors that play a key role in triggering metabolism activation (Amrein et al., 2010, 2014).

Although the cause route of many spoilage defects is still unknown, instrumental methods capable of detecting odorant patterns responsible for perceivable off-odor are of great interest. Moreover, by the implementation of quantitative analytical workflows, quality screening is more objective, and highly-informative chemical analysis can comprehensively cover many needs, e.g., spoilage detection, rancidity, and authentication markers (Cordero et al., 2010; Kiefl et al., 2012; Cialiè Rosso et al., 2018).

Stilo et al. (2021b) developed an effective strategy to detect odorant patterns in selected spoiled hazelnuts showing perceivable defects. A sensory panel screened by flash profiling (FP) (Dairou and Sieffermann, 2002; Delarue and Sieffermann, 2004) Ordu and Akçakoca samples, harvested in 2015 and 2016, at different shelf-life stages. Samples (*n* = 29) were therefore classified into seven sensory classes: “good quality” (*OK* samples) were those eliciting positive attributes and none of the negative attributes arising from the consensus list; “Defected” samples were sub-classified in five different groups based on the predominant off-flavor perceived: *Mold, Mold-rancid-solvent, Rancid, Rancid-stale, Rancid-solvent*, and *Uncoded KO*.

Volatiles were extracted and analyzed with high resolution fingerprinting by HS-SPME followed by GC×GC-TOF MS; 350 untargeted and targeted features were tracked and aligned over all the samples. By unsupervised statistics, *i.e*., hierarchical clustering based on Pearson correlation, a series of informative volatiles showed a strong correlation with *Mold* and *Mold*-*rancid*-*solvent* classes. Volatile fatty acids (hexanoic, heptanoic, octanoic, and nonanoic acid), lactones (γ-hexalactone, δ-heptalactone, γ-heptalactone γ-octalactone, γ-nonalactone), 1-nonanol, and 3-nonen-2-one were distinctive and enabled independent clustering of spoiled hazelnuts from good (*OK*) samples. On the other hand, *OK* samples were connoted by higher amounts of short-chain linear alcohols (2-pentanol and 2-heptanol), butyl ether, butyl benzoate, and 4-heptanone, odorants mainly correlated to positive attributes of *balsamic, fruity*, and *herbal* notes.

**Figure 2:**
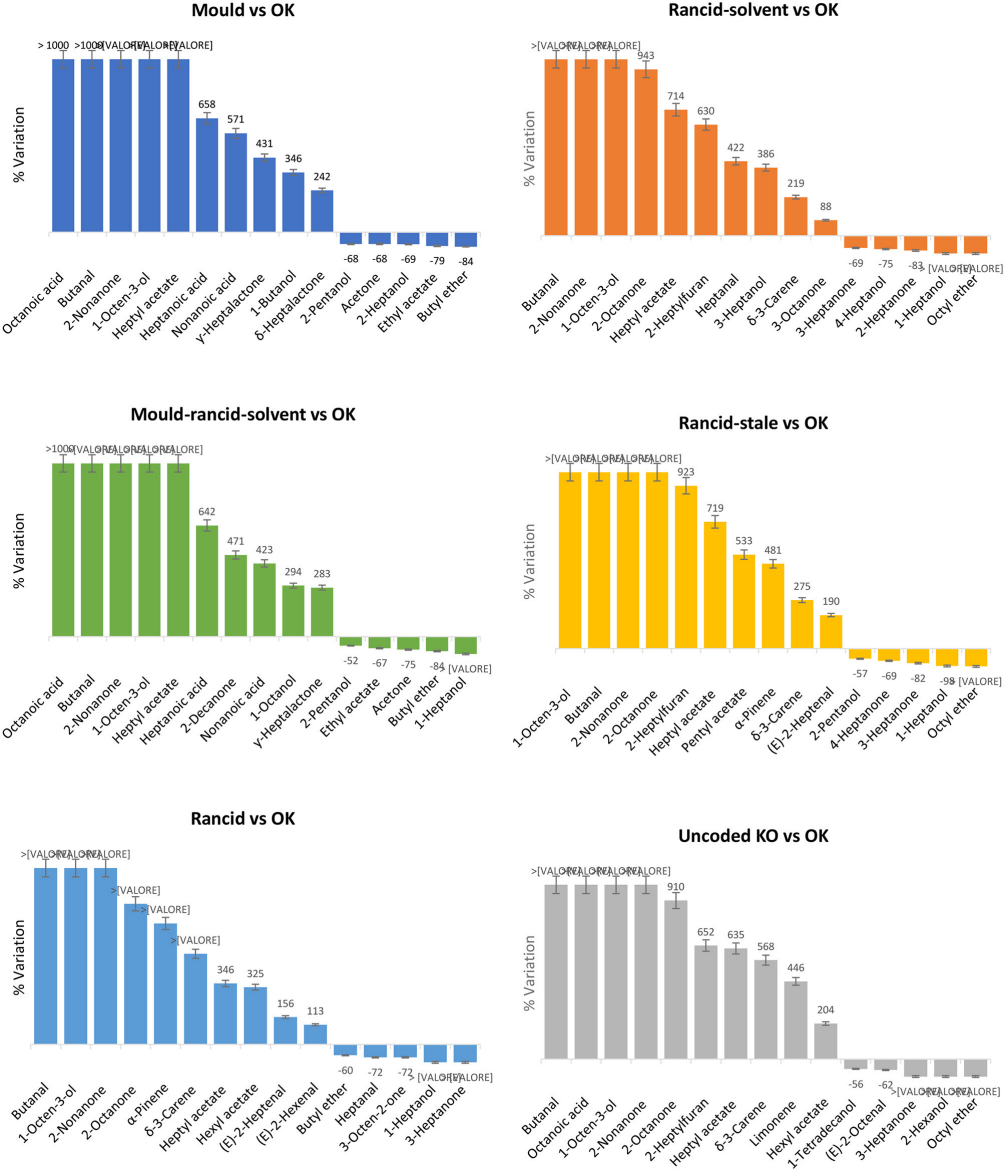
Histograms illustrating the % response ratios for analytes with larger variation between spoiled hazelnuts class-image pairs. OK samples are adopted as reference classes for comparisons. Error bars correspond to ± SD over analytical replicates.

Results on up- or down-regulation of specific volatiles between spoiled and OK samples are illustrated on histograms in Figure 2. *Mold* and *Mold-rancid-solvent samples* were characterized by higher amounts of saturated and unsaturated aldehydes, linear alcohols, and carboxylic acids. Butanal, decanal, and lactones were higher in *Mold*, while pentanoic acid was more abundant in the *Mold-rancid-solvent*. In addition, *Mold-rancid-solvent* class exhibited higher amounts of 3-penten-2-one and 3-octen-2-one, responsible for *earthy* and *musty* notes.

The *Rancid, Rancid-solvent*, and *Rancid-stale* classes had common chemical patterns: higher amounts of linear saturated aldehydes (from C4 to C14) likely informing about the extent of lipid oxidation probably triggered by differential activation of autocatalytic and enzymatic processes (Kinderlerer and Johnson, 1992; Ghirardello et al., 2016; Cialiè Rosso et al., 2018). Moreover, *Rancid-solvent* samples were also connoted by the up-regulation (compared to *OK* samples) of several primary alcohols (1-nonanol, 1-decanol, 1-dodecanol, and 1-tetradecanol), and *Rancid* with higher amounts of 3-penten-2-one and 3-undecanone, while *Rancid-stale* of 2-pentanone and 2-heptanone.

Fatty acids and lactones dominate the differential distribution in *Mold* and *Mold-rancid-solvent* samples: these chemical classes are correlated to the fatty acid degradation promoted by different mold genus (*e.g. Aspergillus, Penicillium, Rhizopus, Fusarium* etc.), developed during post-harvest (Memoli et al., 2017). Lactones are formed by fatty acid hydroxylation in odd or even positions, followed by β-oxidation and chain reduction. As a function of hydroxyl group position, lactonization results in γ-, δ- or ε-lactones (Romero-Guido et al., 2011). Their presence in *moldy* samples, likely contaminated by fungi, is in keeping with literature data; molds produce lactones by enzymatic catalysis with the degradation of hydroxyl fatty acids; when the native substrate is not available, they implement the hydroxylation step (Romero-Guido et al., 2011) to enable lactones formation. Moreover, molds produce lipolytic enzymes (i.e., lipases and esterase) to promote TAGs hydrolysis resulting in a higher amount of high molecular weight lactones produced by higher homologs released from TAGs (Memoli et al., 2017).

Another critical defect is associated with the bug damage, by species like *Palomena prasina* L., *Gonocerus acuteangulatus* G., and *Piezodorus lituratus* F., determining tissue necrosis and decreasing the overall quality of the hazelnut kernel due to off-flavors and bitterness (Singldinger et al., 2018). Singldinger et al. (2018) identified, through a sensory-guided investigation, bitter-tasting diarylheptanoids: asadanin, giffonin P, and other congeners (i.e., (*E*)-7,9,10,13-tetrahydroxy-1,7-bis(2-hydroxyphenyl)hept-9-en-11-one; 4,12,16-trihydroxy-2-oxatricyclo[13.3.1.13,7]-nonadeca-1(18),3,5,7(20),8,15,17-heptaen; 2-(3-hydroxy-2-oxoindolin-3-yl) acetic acid 3-O-6′-galactopyranosyl-2″-(2″oxoindolin-3″yl) acetate and 3-(O-β-d-glycosyl) dioxindole-3-acetic acid) with bitter and astringent taste qualities in *cimiciato*-infected hazelnuts. Although non-volatiles, these sensory active compounds have a detrimental effect on hazelnut sensory quality and might also exert some sensorial synergy with potent odorants responsible for aroma off-notes.

## Potent Odorants and Key-Aroma Compounds Responsible for Hazelnuts Aroma Quality

In the conceptualization of molecular sensory science, i.e., *sensomics* (Schieberle and Hofmann, 2011), key-odorants are those odor active compounds whose amount in the sample exceeds their OT (OAV > 1), and for which omission in aroma recombination experiments confirm the key-role in eliciting the distinctive aroma, i.e., the *aroma blueprint*, of the original product. It has to be noted that recent findings did not exclude that so-called *interferents*, i.e., volatiles with lower or marginal odor activity, might play a role in modulating key-odorants perception while contributing to the overall aroma (Charve et al., 2011; Cordero et al., 2019; Bressanello et al., 2021).

In keeping with these observations, the next paragraphs report major research findings dealing with the identification of potent odorant patterns in raw and roasted hazelnuts. By literature selection, sensory-driven investigations were prioritized because of the intrinsic biological validation they offer concerning the complex phenomenon of olfactory perception.

### Raw Hazelnuts Aroma

Raw hazelnut aroma elicits some specific notes as reported by sensory experiments (descriptive sensory analysis – DSA and quantitative descriptive analysis – QDA): *fruity, fatty, nutty, green, rancid, citrus-like, earthy, flowery, malty, popcorn*-*like, potato*-*like, sour, woody*, and *phenolic* (Burdack-Freitag and Schieberle, 2010; Alasalvar et al., 2012a).

These odor notes were explored by Alasalvar et al. (2003a, 2010, 2012a) in many different cultivars grown in Turkey (Aci, Cavcava, Çakildak, Foşa, Ham, Incekara, Kalinkara, Kan, Karafindik, Kargalak, Kuş, Mincane, Palaz, Sivri, Tombul, Uzunmusa, Yassi Badem, and Yuvarlak Badem). By correlating DSA profiles, GC-O results, and quantitative data on chemical patterns (Alasalvar et al., 2003a), the role of some potent odorants eliciting characteristic aroma was established. However, without the validation step by recombination and omission experiments, it was not possible to define key-aroma compounds (Dunkel et al., 2014) over the group of odorants.

The application of sensomic concepts was successful in this direction, Burdack-Freitag and Schieberle (2010, 2012), Kiefl and Schieberle (2013), and Kiefl et al. (2013) unraveled the aroma code of hazelnuts (raw and roasted) by focusing on different cultivars/origins. They covered the Italian production by studying Tonda Gentile Romana (Lazio, Italy), Tonda Gentile Trilobata (Piemonte, Italy), Tonda Giffoni (Campania, Italy), and Turkey high-quality blend from the Akçakoca region.

Burdack-Freitag and Schieberle (2010, 2012) pre-screened potent odorants by GC-O, performed as aroma extract dilution analysis (AEDA). The distillate/extract obtained by solvent-assisted flavor evaporation (SAFE) of raw hazelnuts extracts, was further fractionated in a neutral-basic fraction (NBF) and the acidic fraction (AF). In the NBF, 37 odor-active zones were detected with a flavor dilution factor (FD) range of 4–4096. Six odorants dominated for their prevalence in the SAFE extracts screening: 2-methoxy-3-isopropylpyrazine elicited an intense *bell pepper-like* odor; 5-methyl-4-heptanone had a *fruity hazelnut-like* note; 2-methoxy-3,5-dimethylpyrazine showed an *earthy* odor; ethyl 2-methylbutanoate, with a *fruity* note; 5-methyl-(*E*)-2-hepten-4-one with *fruity, hazelnut*-*like* aroma; and 2-methoxy-3-isobutylpyrazine showing a *bell pepper-like* attribute. Interestingly, the *fruity hazelnut-like* ketone, 5-methyl-4-heptanone, was characterized for the first time in raw hazelnuts: its very low OT (i.e., 0.05 μg/L in water) is comparable to that of the *nutty* ketone, 5-methyl-(*E*)-2-hepten-4-one (i.e., filbertone).

High relevance was also confirmed for linalool, (*E*)-β-damascenone, and 4-methylphenol (*flowery, boiled apple-like*, and *smoky* notes respectively). Within the acidic fraction, acetic acid (*sour*), 2- and 3-methylbutanoic acid (*sweaty*), and 4-hydroxy-2,5-dimethyl-3(*2H*)-furanone (*caramel*-*like*) were identified.

Accurate quantification by stable isotope dilution analysis (SIDA) (Burdack-Freitag and Schieberle, 2012) identified, within odorants screened by GC-O and AEDA, those exceeding their OT (i.e., OAV > 1). They are listed in Table 6; among them, in raw hazelnuts linalool, 5-methyl-4-heptanone, and 2-methoxy-3,5-dimethylpyrazine showed OAVs > 100 likely dominating the overall aroma of raw kernels. An aroma recombinant including all odorants with OAV >1 conferred a quite high similarity to the aroma profile of raw hazelnuts, with *fruity-nutty, fatty, popcorn-like, flowery, bell-pepper-like, malty*, and *potato-like* considered almost equivalent to the natural aroma profile.

**Table 6:** Key-aroma compounds are defined through the sensomics approach in raw and roasted hazelnut samples from cultivars Tonda Gentile Trilobata (G), Tonda Gentile Romana (R), and Akçakoca blend (A).

**Aroma compound**	**Aroma quality**	**OAV** **(raw R)^**a,b**^**	**OAV** **(roasted R)^**a,b**^**	**OAV** **(raw G)^**b**^**	**OAV** **(roasted G)^**b**^**	**OAV** **(raw A)^**b**^**	**OAV (roasted A)^**b**^**
(*E,E*)-2,4-Decadienal	Deep-fried	<1	7	<1	1	8	6
(*E,E*)-2,4-Nonadienal	Deep-fried	5	6	<1	1	<1	1
(*Z*)-2-Nonenal	Fatty	1	300	\	\	\	\
(Z)-2-Octenal	Fatty	<1	2	\	\	\	\
2,3-Butanedione	Buttery	<1	85	\	\	\	\
2,3-Diethyl-5-methylpyrazine	Musty, nutty	9	266	9	204	9	103
2,3-Pentanedione	Buttery	<1	1,140	\	\	\	\
2-Acetyl-1,4,5,6-tetrahydropyridine	Caramel	46	229	46	219	46	81
2-Acetyl-1-pyrroline	Popcorn-like	13	470	24	599	24	308
2-Acetyl-3,4,5,6-tetrahydropyridine	Roasted, caramel	36	184	36	132	36	36
2-Furfurylthiol	Coffee-like	4	47	8	8	8	8
2-Methoxyphenol	Phenolic, smoky	<1	2	\	\	\	\
2-Methylbutanal	Malty	<1	36	\	\	\	\
2-Phenylacetaldehyde	Green, floral	<1	46	<1	48	<1	41
2-Propionyl-1-pyrroline	Popcorn-like	11	168	22	243	22	113
2-Thenylthiol	Coffee-like	<1	27	\	\	\	\
3-(Methylthio)-propanal	Cooked potato	13	45	\	\	\	\
3-(Methylthio)propionaldehyde	Musty, earthy	15	620	15	442	15	464
3,5,5-Trimethyl-2(5H)-furanone	Seasoning-like	<1	2	\	\	\	\
3,5-Dimethyl-2-ethylpyrazine	Burnt, roasted	1	25	1	11	1	11
3-Ethyl-2,5-dimethylpyrazine	Burnt, roasted	<1	3	<1	2	<1	1
3,7-Dimethylocta-1,6-dien-3-ol	Citrus, floral	12	12	12	12	12	12
3-Methyl-4-heptanone	Fruity, nutty	218	81	126	143	93	91
3-Methylbutanal	Malty	7	1,330	\	\	\	\
3-Methylbutanoic acid	Sour, sweaty	2	8	1	4	1	3
4-Hydroxy-2,5-dimethyl-3(2H)-furanone	Caramel	<1	89	1	79	1	45
4-Hydroxy-3-methoxybenzaldehyde	Vanillic, sweet	<1	4	<1	2	<1	1
5-Methyl-(*E*)-2-hepten-4-one	Nutty, fruity	2	60	2	130	2	71
Acetic Acid	Sour	3	8	\	\	\	\
Dimethyl trisulfide	Sulfurous, cabbage	<1	84	<1	1	8	6
Hexanal	Green	5	5	\	\	\	\
Octanal	Fatty, soapy	3	28	\	\	\	\

*OAV determined after roasting samples at 200°C for 9 min by Burdack-Freitag and Schieberle (2010) and at 160° for 23 min by Kiefl and Schieberle (2013). (a) OAV from Burdack-Freitag and Schieberle (2010); (b) OAV from Kiefl et al. (2013)*.

Kiefl and Schieberle (2013) and Kiefl et al. (2013) further explored the aroma code of hazelnuts by extending the investigation to high-quality cultivars from the two major production areas (Turkey and Italy). Additional odorants, with high OAVs, characterizing raw hazelnuts were identified in 2-propionyl-1-pyrroline, 2-acetyl-1,4,5,6-tetrahydropyridine, and 2-acetyl-3,4,5,6-tetrahydropridine all eliciting *popcorn-like, roasty* notes.

Sensomic studies on hazelnut sensometabolome (Kiefl, 2013), inspired the definition of a performance parameter known as Limit of Odor Activity Value (LOAV), defined as the ratio of the respective OT and the analytical limit of quantification (LOQ). This concept, applied to aroma compounds of Tonda Gentile Trilobata hazelnuts, enabled the evaluation of the method's ability in detecting all key odorants screened by GC-O and AEDA. In particular, in the reference study (Kiefl et al., 2013), authors were able to quantify 30 potent odorants by SIDA with GC×GC-TOF MS, although just 15 of them achieved a LOAV ≥ 1, meaning that the method was unable to assign the appropriate relevance (i.e., key-odorant ranking) to some analytes present at a sub-ppb level. Examples are 2-isopropyl-3-methoxypyrazine, eliciting *green bell pepper*-*like* notes that showed a LOAV = 0.02 and 2-acetyl-1-pyrroline with a *roasty popcorn*-*like* smell, with a LOAV = 0.04.

### Roasted Hazelnuts Aroma

While raw hazelnuts are characterized by a general weak aroma (Kiefl and Schieberle, 2013), the roasting process enhances many existing odor notes (e.g., *nutty-fruity, sweet-caramel*, and *malty)* by triggering several reactions on non-volatile precursors and primary metabolites, while generating new, yet intense, sensations like *roasty, pop-corn like*, and *coffee*-*sulfuric* (Kiefl and Schieberle, 2013).

The chemical reactions triggered by the thermal treatment produces common chemical patterns in many foods; Dunkel et al. (2014) for example, revising the combinatorial odor codes of many foods, identified a strong network structure based on processing technologies (e.g., dry-roasting, fermentation, etc.). Volatiles patterns and odor codes of dry thermally processed foods (roasted, deep-fried, baked) have in common many traits due to the activation of the Maillard reaction (Hofmann and Schieberle, 1998; Van Boekel, 2006; Göncüoglu Taş and Gökmen, 2017), which occurs between reducing sugars and amino acids, and sugars degradation (i.e., caramelization).

As an example, alkyl-pyrazines, characterized by low odor thresholds for humans (e.g., 0.007–0.018 μg L−1 in water), are responsible for the *earthy* and *baked potato*-*like* odors; of them, 2-ethyl-3,5-dimethylpyrazine, 2-ethenyl-3,5-dimethylpyrazine, and 2,3-diethyl-5-methylpyrazine are frequently detected as KFOs in thermally processed foods such as for example, roasted coffee (Blank et al., 1992), French fries (Grosch, 2001), chocolate (Schnermann and Schieberle, 1997), cocoa (Frauendorfer and Schieberle, 2008; Magagna et al., 2018), and peanuts (Chetschik et al., 2010).

In hazelnuts, the complex reaction framework of Maillard reaction, arising by the intersection of many pathways, generates key-odorants impacting on hazelnuts' aroma. They are alkyl methoxy-pyrazines (e.g., *roasty*, 3,5-dimethyl-2-ethylpyrazine/ *earthy;* 2,3,5-trimethylpyrazine*/earthy;* 2,3-diethyl-5-methylpyrazine*/earthy;* 2-isopropyl-3-methoxypyrazine*/ pea-like and green pepper-like*); Strecker aldehydes (2- and 3-methylbutanal/*malty*; phenylacetaldehyde/*flowery* and *honey-like*); ketones (2,3-butanedione and 2,3-pentanedione/*buttery*; 3-methyl-4-heptanone/*fruity* and *nutty*); and heterocycles formed by deoxyosones dehydration in presence of ammonia (e.g., 4-hydroxy-2,5-dimethyl-3(2H)-furanone/*caramel*-*like* and *sweet*; 2-acetyl-1-pyrroline and 2-propionyl-1-pyrroline/*popcorn-like, roasty*) (Alasalvar et al., 2004; Seyhan et al., 2007; Burdack-Freitag and Schieberle, 2012; Kiefl and Schieberle, 2013). Unpleasant notes generated by thermal reactions are those elicited by alkyl-pyridines generally connoted by *burnt* and *astringent* aroma (Van Boekel, 2006).

The role of roasting was the object of many studies aimed at a better understanding of optimal conditions responsible for the generation of pleasant aroma, crunchy texture, and color (Saklar et al., 2001, 2003; Alasalvar et al., 2003a; Alamprese et al., 2009; Cordero et al., 2010; Ciarmiello et al., 2013; Donno et al., 2013; Kiefl and Schieberle, 2013; Belviso et al., 2017; Artik et al., 2021). It was demonstrated that cultivar/origin and storage conditions along shelf-life had a great impact on volatiles generation. Nicolotti et al. (2013a) compared volatiles fingerprints from industrially roasted hazelnuts (Tonda Gentile Trilobata – Piedmont Italy and Ordu – Turkey) to those obtained in lab-scale with specific time/temperature profiles. Authors identified robust markers of roasting namely 5-methylfurfural, 1(H)-pyrrole, furfuryl alcohol, 1(H)-pyrrole-2-carboxaldehyde, 1-hydroxy-2-propanone, dihydro-2(3H)-furanone, acetic acid, pyridine, furfural, pyrazine, and several alkyl-pyrazines; and related indices/ratios whose % increment along roasting kinetic showed great stability among cultivars 5-methylfurfural/ 2,5-dimethylpyrazine; 5-methylfurfural/2-methylpyrazine; and 2,5-dimethylpyrazine/2,3-dimethyl-pyrazine. Interestingly, the key-odorant 5-methyl-(E)-2-hepten-4-one showed a strong cultivar/origin-related increment with Italian Tonda Gentile Trilobata showing an early increase of this potent odorant at mild roasting conditions.

The application of sensomics to roasted hazelnuts unrevealed their aroma blueprint; studies are indicating the presence of some *generalist* key-odorants in combination with others with a more distinctive and peculiar impact on the overall perception (i.e., *individualists*) (Dunkel et al., 2014).

Burdack-Freitag and Schieberle (2010, 2012) for Tonda Gentile Romana hazelnut paste validated the pre-eminent role of several key-odorants here listed in descending order of OAV: 3-methylbutanal/malty/OAV 1330; 2,3-pentanedione/*caramel*/OAV 1140; 2-acetyl-1-pyrroline/*popcorn-like*/OAV 360; (*Z*)-2-nonenal/*tallow*/OAV 300; dimethyl trisulfide/*sulfury, cabbage*/OAV 164; 2-furfurylthiol/*coffee*-*like*/OAV 86; 2,3-butanedione/*buttery*/OAV 85; 4-hydroxy-2,5-dimethyl-3(2H)-furanone/*caramel*/OAV 77; 5-methyl-4-heptanone/*nutty, fruity*/OAV 66; 3-(methylthio)propanal/*cooked potato*/OAV 45; 2-methylbutanal/*malty*/OAV 36; octanal/*soapy*/OAV 28; 2-thenylthiol/*coffee-like*/OAV 27; 2-ethyl-3,5-dimethylpyrazine/*roasted potato*/OAV 21; 5-methyl-(*E*)-2-hepten-4-one/*nutty, fruity*/OAV 13; (*E,E*)-2,4-nonadienal/*deep-fried*/OAV 11; (*E,E*)-2,4-decadienal/*deep-fried*/OAV 10.

Later, Kiefl and Schieberle (2013) and Kiefl et al. (2013), extended the knowledge about high-quality hazelnuts aroma blueprint by elucidating the role of several additional odorants all present at OAVs > 1. They were: 2,3-diethyl-5-methylpyrazine/*earthy, roasty*; 2-acetyl-3,4,5,6-tetrahydropyridine/*popcorn*-*like*, roasty; phenylacetaldehyde/*honey, flowery*; 2-propionyl-1-pyrroline/*popcorn*-*like, roasty*; 4-hydroxy-3-methoxybenzaldehyde/*vanillic, sweet*; and dimethyl trisulfide/*sulfury*.

Moreover, by applying sensomic principles on roasting kinetics (Kiefl et al., 2012; Kiefl and Schieberle, 2013), further insights on the hedonic role of odorant patterns were added. Hazelnuts were submitted to lab-scale roasting in a ventilated oven at 160°C for 12, 23, or 30 min. Cultivars/origin selected were Tonda Gentile Trilobata (G) from Piedmont, Italy; Tonda Gentile Romana (R) from Lazio, Italy; Tonda Giffoni (Gi) from Campania, Italy; Akçakoca blend from Turkey.

Sensory tests were conducted with a trained panel with 24 judges (details in Kiefl and Schieberle, 2013). A QDA and projective mapping experiments were performed on raw and 23 min. roasted samples to evaluate similarities and differences among samples. QDA evaluated the aroma intensity of eight attributes (*coffee-like, sulfury; nutty, fruity; smoky, phenolic; malty; sweet, caramel-like; roasty, popcorn-like; fatty; earthy, green*) by a seven-point scale in a range between 0-3. For the projective mapping, panelists were instructed as follows: “*Two samples should be placed very near if they seem identical, and two samples should be placed distant to one another if they seem different to you; this should be done according to your own criteria; do not hesitate to express strongly the differences you perceive by using the most part of the screen (total space)*” (Kiefl and Schieberle, 2013).

**Figure 3:**
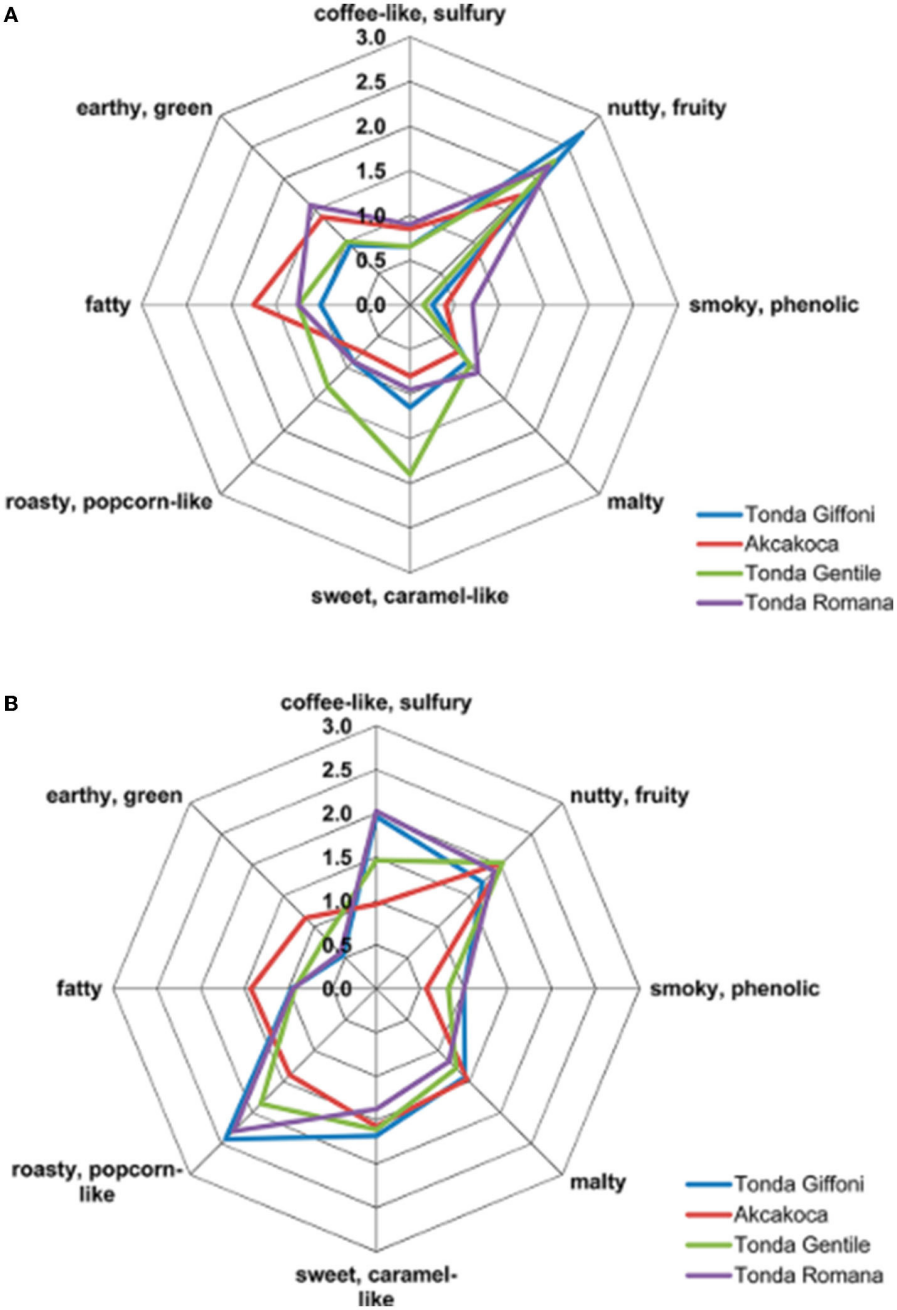
Qualitative sensory analysis results illustrated as spider graphs and corresponding to four hazelnuts cultivars/blends as raw **(A)** and roasted **(B)** [23 min at 160°C] kernels. Figure from Kiefl and Schieberle (2013).

Aroma profiles of raw hazelnuts, as shown by the spider-graph resulting from the QDA (Figure 3A), were very similar, as also stated by other authors (Seyhan et al., 2007). On the other hand, roasted samples showed some meaningful intensity differences. The roasted Tonda Gentile Trilobata from Piedmont (G), reported weaker *coffee-like* and *roasty* odor notes (Figure 3B) accompanied by an intense *nutty* perception. However, by comparing concentration profiles for key-odorants among all analyzed samples (Table 4), sensory differences and odorant patterns could not be easily correlated.

Some analytes, most sensitive to roasting conditions (Kiefl et al., 2012; Nicolotti et al., 2013a), had a 100-fold increase from raw (0) to 30 min; They were identified as: 5-methyl-(*E*)-2-hepten-4-one; 2-acetyl-1-pyrroline; 2-propionyl-1-pyrroline; 2-acetyl-1,4,5,6-tetrahydropyridine; 2-acetyl-3,4,5,6-tetrahydropyridine; 2-acetylpyridine,3,6-dimethyl-2-ethylpyrazine; 3-(methylthio)propionaldehyde; 2-phenylacetaldehyde; 3,5-dimethyl-2-ethylpyrazine; and 2,3-diethyl-5-methylpyrazine.

Projective mapping experiments showed great correlation, as a primary variable, to the roasting degree, resulting in three main clusters defined by raw hazelnuts, mild-to-optimal roasting, and over-roasting conditions. Moreover, some samples were classified into separate groups. Assessors intuitively ordered samples according to the roasting degree along the first dimension (i.e., horizontal axis) of the project map while using the second dimension (i.e., the vertical axis) to discriminate hazelnuts by a hedonic scale. Further experiments, recombining odorant patterns for optimally roasted hazelnuts in a sunflower oil medium, confirmed the pre-eminent role of identified key odorants and suggested some synergies and suppression phenomena between them.

The perceptual pattern obtained with aroma recombinants shared a high degree of similarity with those obtained by real samples. Authors stated that eight key-odorants were fundamental to reconstruct the hazelnut aroma blueprint: 3-methyl-4-heptanone elicits the weak *nutty* aroma of raw hazelnuts, while the pattern of 5-methyl-(*E*)-2-hepten-4-one, 2-acetyl-1-pyrroline, 2-propionyl-1-pyrroline, 3,6-dimethyl-2-ethylpyrazine, 3,5-dimethyl-2-ethylpyrazine, 2,3-diethyl-5-methylpyrazine elicits a *nutty, roasty* aroma; high amounts of 2-furfuryl mercaptan impart *roasty* and *coffee*-*like* odor in over-roasted samples.

Accurate quantification of aroma compounds by SIDA, or suitable approaches (Sgorbini et al., 2019), combined with GC×GC-TOF MS proved to be highly effective to decode the aroma blueprint at a molecular level due to the very low LOAVs achievable (Kiefl et al., 2013; Nicolotti et al., 2013b). However, besides the distinctive patterns of key odorants that clearly evoke hazelnut aroma qualities, to date researchers did not find any other volatile capable of consistently explaining hedonic differences across different cultivars. Sensory-oriented strategies, accompanied by high-resolution separations (i.e., multidimensional analytical approaches) and data mining represent the ultimate solution to unravel the combinatorial code of olfaction behind food sensory perception.

The role of so called *interferent* volatiles (Cordero et al., 2019) should be better elucidated since they can modulate odorant pattern perception, enabling differential activation of the Receptor Code. Moreover, the presence of chiral odorants in enantiomeric excess or distinctive ratios might influence the hedonic profile; for example, 5-methyl-(*E*)-2-hepten-4-one (i.e., filbertone) enantiomers are characterized by different odor qualities and OTs. The next paragraph reports some insights on filbertone chiral recognition and functional properties.

### Filbertone as Hazelnuts Individualist Key-Odorant

The molecule configuration is crucial in determining its aroma perception: enantiomers may differ in the aroma intensity, as it is the case of menthol and camphor, or even in the flavor itself, as it is for 3-methylthiobutanal (i.e., methional) where the (*R*)-configured molecule elicits the typical odor of *cooked potatoes*, while the (S)-configured stereoisomer is odorless (Weber and Mosandl, 1997; Zawirska-wojtasiak, 2006). Such characteristic is fundamental with hazelnuts too since filbertone (i.e., 5-methyl-(*E*)-2-hepten-4-one), the key-odorant contributing to the *nutty* aroma (Jauch et al., 1989; Alasalvar et al., 2004; Burdack-Freitag and Schieberle, 2012; Kiefl et al., 2013), might be present as *R* or *S* enantiomer(s) on the chiral center on C5 (Puchl'ová and Szolcsányi, 2018).

Filbertone amounts greatly vary among cultivars, higher levels were reported for Tonda Gentile Trilobata cultivar independently by harvest area (Piedmont Italy vs. Georgia) (Cialiè Rosso, 2020), justifying the fact that this cultivar is particularly appreciated by consumers and defined as the *gold standard* in the confectionery industry.

Jauch et al. (1989) were the first that effectively discriminated by enantioselective GC (ES-GC) the *R*- and *S*-filbertone while also describing marked olfactory differences in terms of odor intensity and quality, with the *S-* enantiomer characterized by *metallic, fatty*, and *pyridine* perceptions, while the *R-* one by *buttery* and *chocolate-like* notes; moreover, the *R-* enantiomer has a 10-fold lower odor threshold. The *R*- and *S*- enantiomers are not equally abundant in the kernel: Ruiz del Castillo et al. (2003) investigated the enantiomeric distribution in both raw and roasted hazelnuts obtaining between 70 and 90% of enantiomeric excess (*ee*) for the *S*-filbertone depending on the variety regarding raw hazelnuts (e.g., Turkish hazelnuts showed 54–56% *ee*, whereas Italian ones revealed 62–63% *ee*) (Jauch et al., 1989). Roasted samples, instead exhibited an *ee* of only 17% for the *S*-enantiomer. Nevertheless, the filbertone concentration increased by 35-fold during roasting. The differential increase of the R-enantiomer during roasting could be likely due to a *thermal* pathway (Güntert et al., 1991; Blanch and Jauch, 1998) whose precursors are still unknown.

## Correlations Between Primary Metabolites and Aroma Compounds: The Concept of *Aroma Potential*

The concept of *aroma potential* was firstly introduced by Cialiè Rosso et al. (2018) in a study focused on hazelnut volatilome evolution along with shelf-life. The concept arose by the observation that post-harvest drying conditions appeared fundamental to inactivate exogenous and endogenous enzymes, providing more stable kernels throughout their shelf-life, independently of storage conditions (e.g., atmosphere composition, temperature, and time). Volatile patterns evolution indicated that lipid oxidation and spoilage occurred more decisively on those samples exposed to a less efficient drying (i.e., sun-drying *vs*. industrial drying at low temperatures), stored at normal atmosphere and ambient temperature.

Researchers analyzed volatiles patterns from hazelnuts roasted at lab-scale (160°C-15 min) after storage in specified conditions (see Section Effect of Post-Harvest and Storage on Volatilome Signatures) (Cialiè Rosso et al., 2018). Secondary products of hydroperoxide cleavage (i.e., linear saturated aldehydes –from C5 to C10-, unsaturated aldehydes - (*E*)-2-heptenal, (*E*)-2 octenal ad (*E*)-2 decenal, short-chain fatty acids - pentanoic, octanoic, and nonanoic acid, and linear alcohols – from C5 to C8) were close to the method LOD in freshly roasted hazelnuts (T0) or in those stored in a modified atmosphere. An increasing trend over storage time was also observed for kernels stored in a normal atmosphere as a function of temperature (5 or 18°C), as additional stress factors.

Of interest was the trend for some key-odorants responsible for the *malty* and *buttery* (2- and 3-methylbutanal, 2,3-butanedione, and 2,3-pentanedione), *earthy* (methylpyrazine, 2-ethyl-5-methyl pyrazine, and 3-ethyl-2,5-dimethyl pyrazine) and *caramel-like* (2,5-dimethyl-4-hydroxy-3(2H)-furanone) and *musty* (acetyl pyrrole) notes. Storage at lower temperatures (5°C) and low-temperature drying preserved their amount in both cultivars/origin (i.e., Tonda Gentile Romana and Ordu) along with shelf-life. The authors argued that there was a fairly stable distribution of their precursors along with shelf-life.

These results suggested the correlation of non-volatile precursors with potent odorants characterizing roasted hazelnut aroma; as for lipid fraction, degradation reactions and kernel viability would have had an impact on primary metabolites known to form under roasting conditions key-aroma substances.

The Pearson correlation coefficient (*r*) was adopted to evaluate positive or negative correlations between primary metabolites and key-informative volatiles. The study applied advanced fingerprinting strategies combining untargeted and targeted features information from GC×GC-TOF MS analyses of hazelnut polar extracts followed by oximation-silylation on amino acids, reducing sugars, polyols, and organic acids (Cialiè Rosso et al., 2020, 2021). Samples analyzed were from Tonda Gentile Trilobata, Tonda Gentile Romana, and Ordu.

**Figure 4:**
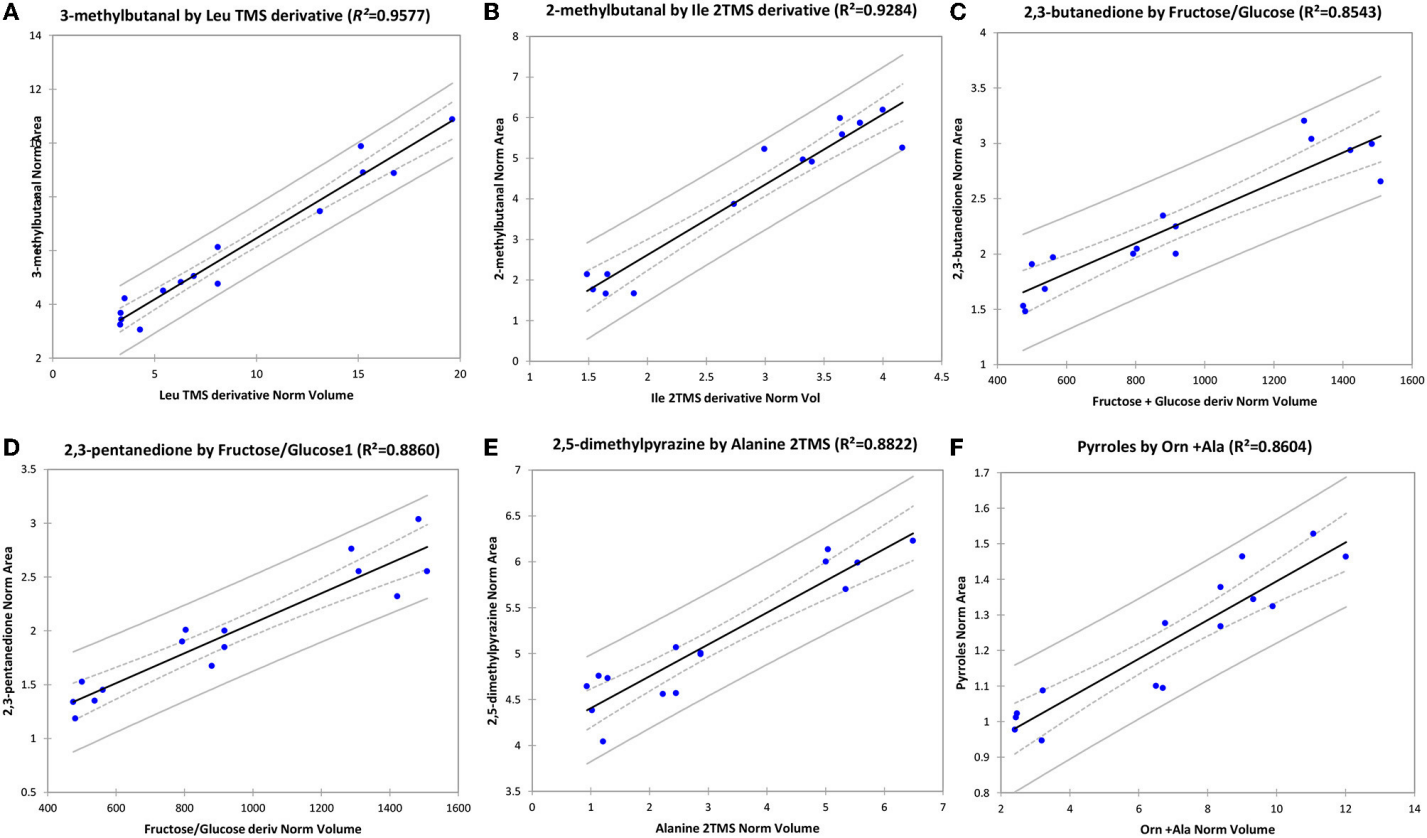
Linear regression functions (confidence interval 95%) between primary metabolites and/or precursors with corresponding key-odorants in roasted kernels. **(A)** 3-Methylbutanal with Leucine (R^2^ 0.9577), **(B)** 2-Methylbutanal and Isoleucine (R^2^ 0.9284), **(C)** 2,3-Butanedione and **(D)** 2,3-Pentanedione and the sum of fructose (*syn*- and *anti*- forms) and glucose (glucopyranose and glucose) (R^2^ 0.8543 and 0.8860), **(E)** 2,5-Dimethylpyrazine and Alanine (R^2^ 0.8822), and **(F)** pyrroles and the sum of Ornithine and Alanine (R^2^ 0.8604) (from Cialiè Rosso et al., 2020).

Results showed good correlations (*p*-values <0.05) within primary metabolites, also indicating that Tonda Gentile Trilobata and Tonda Gentile Romana samples showed a higher amount of some non-volatile precursors and primary metabolites compared to the Ordu blend. On the other hand, interesting correlations were established between primary metabolites and volatiles eliciting aroma qualities. These correlations were further explored and tested for their linearity. The coefficients of determination (*R*^2^) of the linear regression were estimated for the relation between the precursor(s) as the independent variable (*x*) and key-volatiles as the dependent variable (*y*). Figure 4 reports regression functions between 3-methylbutanal and leucine-Leu (*R*^2^ 0.9577), 2-methylbutanal and Isoleucine-Ile (*R*^2^ 0.9284), 2,3-butanedione and 2,3-pentanedione and fructose/glucose derivatives (*R*^2^ 0.8543 and 0.8860), between 2,5-dimethylpyrazine and alanine-Ala (*R*^2^ 0.8822), and pyrroles and the sum of ornithine-Orn and Alanine-Ala (*R*^2^ 0.8604).

Results, although preliminary due to the limited sample set available, posed a solid foundation for the *aroma potential* concept; a comprehensive yet quantitative fingerprinting of hazelnut primary metabolome could be used as an effective tool to predict the aroma potential of hazelnuts (Cialiè Rosso et al., 2018).

## Conclusions and Future Perspectives

Modern investigation approaches inspired by “omics” strategies have the potentials to address most of the challenges posed by the study of complex food metabolome/volatilome in relation to biological phenomena (Miguel et al., 2011; Capozzi and Bordoni, 2013). In the case of hazelnuts, the effect of functional variables at the basis of phenotype expression, reaction to extreme climate events and local pedo-climatic conditions, changes during storage, odor quality, and hedonic profile have been observed, interpreted, and in several cases rationally modeled after effective and reliable exploration of the compositional complexity of entire volatilome.

Sensory guided strategies have identified key-aroma patterns evoking the unique and distinctive hazelnut flavor (Hofmann and Schieberle, 1995). However, it is still underexplored the role of ancillary odorants, i.e., those that do not exceed their odor perception threshold in the sample, that by interacting with ORs in the olfactory epithelium, might modulate the overall aroma perception with effects on hedonic properties (Cordero et al., 2019).

Moreover, efforts are needed to better understand the volatilome expression under the effect of key-functional variables, known to have a negative impact on sensory quality. Besides the autoxidation of lipids, responsible for the generation of potent odorants with unpleasant notes [e.g., (*E,E*)-2,4-nonadienal and (*E,E*)-2,4-decadienal – *deep*-*fried*; *(Z*)-2-octenal and (*Z*)-2-nonenal – *fatty*; hexanal – *green*; octanal – *fatty, soapy*], insights are expected on enzymatic degradation of lipids. The evolution of free and esterified fatty acids along shelf life might have an impact on lipid oxidation stability. A better understanding of lipase/esterase activity could guide targeted actions to inhibit enzymes activity during post-harvest and storage (Cialiè Rosso et al., 2021).

The interconnection between primary and specialized non-volatile metabolites patterns and volatiles generated during storage and industrial processing (i.e., dry-roasting), should be better explored. In this context, interesting outcomes could support the development of predictive models for odorant formation and aroma potential expression (Cialiè Rosso et al., 2018, 2020) to be used as decision-makers at an industry level.

The industrial need for effective solutions to practical problems related to hazelnut quality management urges the adoption of multidisciplinary yet systemic approaches [i.e., *omics* strategies (Wishart, 2008; Ulaszewska et al., 2019)] giving access to a higher level of information closer to a better understanding of complex phenomena (Nanda and Das, 2011).

## Author Contributions

SS: data curation, validation, and writing-original draft, review and editing. FS, MC, and EL: writing review and editing. CB: funding acquisition, conceptualization, supervision, and writing review and editing. NS: project administration, conceptualization, and writing review and editing. GC and GG: conceptualization and writing review and editing. CC: funding acquisition, project administration, conceptualization, supervision, and writing-original draft, review and editing. All authors contributed to the article and approved the submitted version.

## Conflict of Interest

FS was employed by Laemmegroup, a Tentamus Company. NS, GC, and GG are employees of Soremartec Italia srl, a Ferrero Group company. The remaining authors declare that the research was conducted in the absence of any commercial or financial relationships that could be construed as a potential conflictof interest.

## Publisher's Note

All claims expressed in this article are solely those of the authors and do not necessarily represent those of their affiliated organizations, or those of the publisher, the editors and the reviewers. Any product that may be evaluated in this article, or claim that may be made by its manufacturer, is not guaranteed or endorsed by the publisher.
